# Mediation of Age and Thrombectomy Outcome by Neuroimaging Markers of Frailty in Patients With Stroke

**DOI:** 10.1001/jamanetworkopen.2023.49628

**Published:** 2024-01-02

**Authors:** Faysal Benali, Nishita Singh, Joachim Fladt, Tanaporn Jaroenngarmsamer, Fouzi Bala, Johanna M. Ospel, Brian H. Buck, Dar Dowlatshahi, Thalia S. Field, Ricardo A. Hanel, Lissa Peeling, Michael Tymianski, Michael D. Hill, Mayank Goyal, Aravind Ganesh

**Affiliations:** 1 Calgary Stroke Program, Department of Clinical Neurosciences, University of Calgary, Cumming School of Medicine, Calgary, Alberta, Canada; 2 Hotchkiss Brain Institute, University of Calgary, Cumming School of Medicine, Calgary, Alberta, Canada; 3 Mathison Centre for Mental Health Research and Education, University of Calgary, Cumming School of Medicine, Calgary, Alberta, Canada; 4 Department of Radiology, University of Calgary, Cumming School of Medicine, Calgary, Alberta, Canada; 5 Department of Radiology and Nuclear Medicine, Maastricht UMC+, Maastricht, the Netherlands; 6 Neurology Division, Department of Internal Medicine, University of Manitoba, Max Rady College of Medicine, Winnipeg, Manitoba, Canada; 7 Department of Neurology, University Hospital Basel, University of Basel, Basel, Switzerland; 8 Diagnostic and Interventional Neuroradiology Department, University Hospital of Tours, Tours, France; 9 Department of Radiology and Nuclear Medicine, University Hospital Basel, University of Basel, Basel, Switzerland; 10 Division of Neurology, Department of Medicine, University of Alberta, Edmonton, Alberta, Canada; 11 Department of Medicine (Neurology), Neuroradiology Section, The Ottawa Hospital, University of Ottawa, Ottawa Hospital Research Institute, Ottawa, Ontario, Canada; 12 Division of Neurology, University of British Columbia, Vancouver, British Columbia, Canada; 13 Lyerly Neurosurgery, Baptist Neurological Institute, Baptist Health, Jacksonville, Florida; 14 Saskatoon Stroke Program, Royal University Hospital, University of Saskatchewan, Saskatoon, Canada; 15 NoNO Inc, Toronto, Ontario, Canada; 16 Department of Community Health Sciences, University of Calgary, Cumming School of Medicine, Calgary, Alberta, Canada; 17 O’Brien Institute for Public Health, University of Calgary, Cumming School of Medicine, Calgary, Alberta, Canada

## Abstract

**Importance:**

Age is a leading predictor of poor outcomes after brain injuries like stroke. The extent to which age is associated with preexisting burdens of brain changes, visible on neuroimaging but rarely considered in acute decision-making or trials, is unknown.

**Objectives:**

To explore the mediation of age on functional outcome by neuroimaging markers of frailty (hereinafter neuroimaging frailty) in patients with acute ischemic stroke receiving endovascular thrombectomy (EVT).

**Design, Setting, and Participants:**

This cohort study was a post hoc analysis of the Safety and Efficacy of Nerinetide (NA-1) in Subjects Undergoing Endovascular Thrombectomy for Stroke (ESCAPE-NA1) randomized clinical trial, which investigated intravenous (IV) nerinetide in patients who underwent EVT within a 12-hour treatment window. Patients from 48 acute care hospitals in 8 countries (Canada, US, Germany, Korea, Australia, Ireland, UK, and Sweden) were enrolled between March 1, 2017, and August 12, 2019. Markers of brain frailty (brain atrophy [subcortical or cortical], white matter disease [periventricular or deep], and the number of lacunes and chronic infarctions) were retrospectively assessed while reviewers were blinded to other imaging (eg, computed tomography angiography, computed tomography perfusion) or outcome variables. All analyses were done between December 1, 2022, and January 31, 2023.

**Exposures:**

All patients received EVT and were randomized to IV nerinetide (2.6 mg/kg of body weight) and alteplase (if indicated) treatment vs best medical management.

**Main Outcome and Measures:**

The primary outcome was the proportion of the total effect of age on 90-day outcome, mediated by neuroimaging frailty. A combined mediation was also examined by clinical features associated with frailty and neuroimaging markers (total frailty). Structural equation modeling was used to create latent variables as potential mediators, adjusting for baseline, early ischemic changes; stroke severity; onset-to-puncture time; nerinetide treatment; and alteplase treatment.

**Results:**

Among a total of 1105 patients enrolled in the study, 1102 (median age, 71 years [IQR, 61-80 years]; 554 [50.3%] male) had interpretable imaging at baseline. Of these participants, 549 (49.8%) were treated with IV nerinetide. The indirect effect of age on 90-day outcome, mediated by neuroimaging frailty, was associated with 85.1% of the total effect (β coefficient, 0.04 per year [95% CI, 0.02-0.06 per year]; *P* < .001). When including both frailty constructs, the indirect pathway was associated with essentially 100% of the total effect (β coefficient, 0.07 per year [95% CI, 0.03-0.10 per year]; *P* = .001).

**Conclusions and Relevance:**

In this cohort study, a secondary analysis of the ESCAPE-NA1 trial, most of the association between age and 90-day outcome was mediated by neuroimaging frailty, underscoring the importance of features like brain atrophy and small vessel disease, as opposed to chronological age alone, in predicting poststroke outcomes. Future trials could include such frailty features to stratify randomization or improve adjustment in outcome analyses.

## Key Points

**Question:**

To what extent do neuroimaging markers of brain frailty mediate associations between age and postthrombectomy outcomes in ischemic stroke?

**Findings:**

In this cohort study, a post hoc analysis of the Safety and Efficacy of Nerinetide (NA-1) in Subjects Undergoing Endovascular Thrombectomy for Stroke (ESCAPE-NA1) randomized clinical trial, markers of brain frailty on routine computed tomography images (brain atrophy, chronic infarcts, and small vessel disease) mediated 85.1% of the total association of age with 90-day functional outcome after thrombectomy.

**Meaning:**

The findings of this study suggest that neuroimaging markers of frailty are important to consider when performing outcome predictions in clinical practice and when adjusting for primary outcome analyses in future trials, since imbalances in brain frailty features could confound treatment effects.

## Teaser

This cohort study is a post hoc analysis of the Safety and Efficacy of Nerinetide (NA-1) in Subjects Undergoing Endovascular Thrombectomy for Stroke randomized clinical trial to assess how neuroimaging markers of frailty mediate the association of age with postthrombectomy outcomes in patients with ischemic stroke.

## Introduction

Age is a leading, nonmodifiable predictor of 90-day functional outcome after acquired brain injuries like stroke.^1,2^ Endovascular thrombectomy (EVT) is an established treatment for acute ischemic stroke (AIS) due to large vessel occlusion,^3,4,5^ and older age is associated with worse EVT outcomes,^6^ including futile recanalization, symptomatic hemorrhages, and mortality.^6,7,8^

The association of age with functional outcome could partly be explained by differences in “brain reserve” among patients. This concept refers to the redundance of brain networks that determine the presence and severity of symptoms after brain injury, including AIS.^9^ In other words, it determines the amount of injury that can be sustained before reaching a threshold for clinical expression. Brain reserve can diminish over time due to several factors (environmental [eg, smoking], underlying disease [eg, diabetes], or genetic predisposition) that can make the brain frailer or less resilient. This process can occur at a different pace than chronological aging.^10,11,12,13^

Imaging surrogates for brain frailty (ie, lack of brain reserve), easily detectable on noncontrast computed tomography (NCCT), include brain atrophy (cortical and subcortical) and features of cerebral small vessel disease (eg, lacunes, white matter disease [WMD], and chronic infarctions).^14^ Clinical surrogates include cardiovascular comorbidities (eg, diabetes, smoking, hypertension, and hypercholesterolemia), prior stroke, or traumatic brain injury.

The individual features of brain frailty, such as brain atrophy, features of cerebral small vessel disease, or prior stroke, are associated with poor functional outcome after EVT.^15,16,17,18,19,20,21^ However, the extent to which these measures collectively contribute to worse EVT outcomes with age is unknown. The importance of this matter extends beyond stroke and EVT; for instance, brain atrophy is also associated with worse outcomes after traumatic brain injury.^22^

In this study, we explored how neuroimaging markers of frailty (hereinafter neuroimaging frailty), as aggregate constructs, mediated the association between age and 90-day outcome in patients with AIS who underwent EVT within a 12-hour window. Second, we examined the extent of mediation attributable to the combination of neuroimaging and clinical features associated with frailty.

## Methods

This cohort study was a post hoc analysis of the Safety and Efficacy of Nerinetide (NA-1) in Subjects Undergoing Endovascular Thrombectomy for Stroke (ESCAPE-NA1) multicenter, double-blind, randomized clinical trial, which investigated the safety and efficacy of intravenous (IV) nerinetide in patients with AIS due to large vessel occlusion who were undergoing EVT.^23^ Written informed consent was obtained from all participants or their legally authorized representatives. The ethics board at each center and local regulatory authorities approved the study. Between March 1, 2017, and August 12, 2019, participants from 48 acute care hospitals in 8 countries (Canada, US, Germany, Korea, Australia, Ireland, UK, and Sweden) in the ESCAPE-NA1 trial were enrolled and randomized to receive a 2.6-mg/kg of body weight dose of IV nerinetide or placebo in addition to best medical therapy, including IV alteplase if indicated. This study followed the Strengthening the Reporting of Observational Studies in Epidemiology (STROBE) reporting guideline.

In brief, inclusion criteria were (1) age of 18 years or older, (2) baseline National Institutes of Health Stroke Scale (NIHSS) score greater than 5 (scores range from 0 to 42, with higher scores indicating severe stroke), (3) functional independence before the index stroke (Barthel Index for Activities of Daily Living score >90 [scores range from 0 to 100, with higher scores indicating independence in physical functioning]), and (4) <12 hours since the last time the patient was known to be well. Imaging eligibility criteria included (1) large-vessel occlusion (intracranial internal carotid artery, M1 occlusion, or functional M1 occlusion [occlusion of both M2 branches]) on baseline CT angiography, (2) moderate-to-good collaterals (defined as filling ≥50% of middle cerebral artery territory on multiphase CT angiography), and (3) an Alberta Stroke Program Early Computed Tomography Score (ASPECTS) of 5 or more (scores range from 0 to 10, with lower scores indicating more middle cerebral artery regions involved) on baseline NCCT.

### Patient Sample

**Figure 1.**
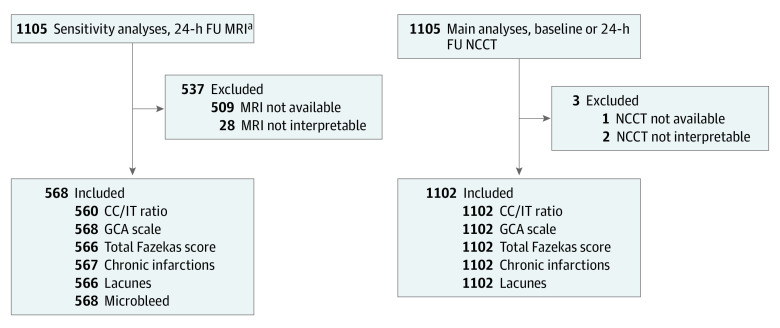
Study Flowchart CC/IT indicates ratio of intercaudate distance to inner table width; FU, focused ultrasound; GCA, global cortical atrophy; MRI, magnetic resonance imaging; NCCT, noncontrast computed tomography. ^a^At least axial fluid-attenuated inversion recovery sequences available.

All patients with interpretable NCCT, including at least axial planes without artifacts (eg, motion or beam hardening) disturbing interpretation of relevant features, were included in the study. Sensitivity analyses included all patients who had interpretable, 24-hour magnetic resonance imaging (MRI), including at least axial fluid-attenuated inversion recovery (see Figure 1 for study flowchart).

### Image Analysis

All imaging data were reviewed by an independent core laboratory blinded to clinical and outcome data. Disagreements were resolved by consensus from a senior reader (A.G.) (see eTable 1 in Supplement 1). All included NCCT images were acquired with a minimum power of 120 kV to 140 kV; 170 mA to 200 mA; 2-second scanning; 5 mm section thickness, with appropriate algorithms for reducing bone artifacts and increasing signal-to-noise ratio; contiguous axial sections from skull base to vertex parallel to the inferior orbitomeatal line, with helical acquisitions; and 3 mm reformats.^23^ Cortical atrophy was assessed by using the global cortical atrophy scale.^24^ This pragmatic, qualitative scale evaluates the degree of cortical atrophy by assessing the width of the sulci and the volume of the gyri (ranging from 0 for no atrophy to 3 for severe atrophy).^24^ Subcortical atrophy was assessed by calculating the ratio of the intercaudate distance to inner table width ratio.^25^ To avoid measurement error due to potential infarct-associated edema, the intercaudate distance was measured on the contralateral hemisphere (ie, hemi- intercaudate distance) and then multiplied by 2 (eFigure 1 in Supplement 1). The hemi-intercaudate distance is defined as the minimum distance between the caudate head and the septum pellucidum at the level of the foramen of Monro and has been used previously to assess subcortical atrophy in patients with stroke.^26,27^ Periventricular and deep white matter lesions were visually assessed by applying the Fazekas scale^28^ for both locations. This scale evaluates the degree of WMD in both periventricular and deep locations, with the score for each location ranging from 0 (no WMD) to 3 (large confluent areas or hypodensities extending to the cortex), combined to generate a total WMD-burden score. We adopted the Fazekas scale to NCCT, which has been done before.^29,30,31^ Assessments again involved the contralateral hemisphere to avoid confounding by acute stroke. Old infarcts were defined as any cortical or subcortical area of tissue loss exceeding an axial diameter of 15 mm. Lacunes were defined as round or ovoid lesions filled with cerebrospinal fluid ranging from 3 mm to 15 mm with a hyperintense rim on fluid-attenuated inversion recovery sequences. For NCCT analyses, any round or ovoid lesion with a cerebrospinal fluid signal ranging from 3 mm to 15 mm was considered a lacuna.

Additional sensitivity analyses were performed assessing aforementioned scales and measures in patients who had 24-hour MRI. In those assessments, we added the number of microbleeds, presence of superficial siderosis, and enlarged perivascular spaces. Microbleeds and superficial siderosis were detected on blood-sensitive sequences (preferably T2*-weighted gradient echo; if not available, then susceptibility-weighted imaging or regular T2 sequences) and were defined as any round or ovoid lesion that caused blooming artifacts. Superficial siderosis (chronic blood products in the superficial cortex under the pia mater, caused by chronic consequences of repetitive subarachnoid hemorrhages) manifested as linear hypointensities over the cortex.^14^ Enlarged perivascular spaces were assessed on T2 sequences using the Edinburgh scale and were defined as ovoid or linear configurations of cerebrospinal fluid signal, distinguished from lacunes by lack of a hyperintense rim and because enlarged perivascular spaces are less than 3 mm.^14^

### Clinical Variables

All participants had standard assessments of demographic characteristics, medical history, laboratory values, and stroke severity (NIHSS score) at baseline. The functional outcome (modified Rankin Scale [mRS]) was assessed by trained personnel at 90 days after stroke who were unaware of treatment-group assignment (ie, nerinetide vs control). The mRS ranges from 0 to 6, with 0 indicating perfect health without symptoms and 6 indicating death.

### Statistical Analysis

Analyses were performed between December 1, 2022, and January 31, 2023. Baseline characteristics were reported using descriptive statistics. Categorical variables were reported as frequencies and percentages, whereas quantitative variables were reported as medians and IQRs. Comparisons of baseline characteristics were based on a dichotomization of age (according to the median 71 years). For categorical variables, we used χ^2^ tests when expected counts were greater than 5 and Fisher exact tests when the expected counts were less than 5. For continuous variables, we used the Wilcoxon rank sum test.

To analyze the mediating effect of neuroimaging frailty, we used structural equation modeling (SEM), a linear regression model framework, built to include theoretical, nonobserved (ie, latent) variables in regression models.^32^ In this study, we created a latent variable,^33^ a theoretical construct built from several measurable variables. Using SEM, we could include latent variables, as well as measurable variables, simultaneously in the same construct, with each observed variable linked to the latent variable through a linear equation. This way, conclusions drawn on a model level would be more valid compared with traditional multivariate regression models. Major strengths of using SEM^32^ are the ability to take into account unmeasured factors and the suitability for data with measurement errors, variability (eg, interrater variations), or incomplete measurements (ie, not all variables need to be available in all participants).

**Figure 2.**
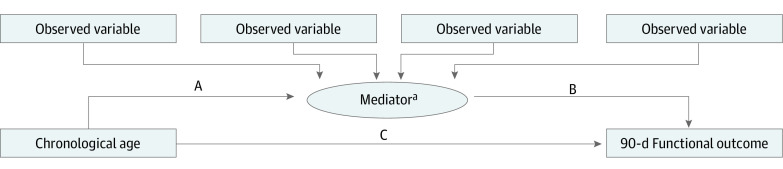
Causal Hypothesis Diagram The direct effect is defined as pathway C, and the indirect effect is defined as pathways A × B. Rectangles represent observed variables; ovals represent latent variables. Linear equations are built by using arrows between the variables; 90-day functional outcome was included as an ordinal variable following the full categorical scale (ie, modified Rankin Scale, which ranges from 0 to 6, with 0 indicating perfect health without symptoms and 6 indicating death). ^a^This model is repeated 2 times; a different model is created for each latent variable as a different mediator (ie, neuroimaging frailty and composite frailty).

Structural equation modeling was then used for several mediation analyses, in which we included the latent variable as a potential mediator of the effect of age (ie, the independent variable) and the 90-day mRS (ie, the dependent variable). Mediation analyses were performed to better understand the proportion of the mediating effect on the association between age and 90-day outcome. As opposed to a direct association between an independent variable (chronological age) and a dependent variable (90-day mRS), a mediation model proposes that the independent variable first influences the mediator, which in turn influences the dependent variable (Figure 2). All models were adjusted for the administration of nerinetide and/or alteplase, the onset-to-puncture time, NIHSS score at baseline, and ASPECTS at baseline.

We proposed 2 models, each of them including a different latent variable. The first model included neuroimaging frailty as a mediator, which was created by the following observed variables, assessed on NCCT: cortical atrophy, subcortical atrophy, total WMD burden, number of lacunes, and number of chronic infarctions. The second model included composite frailty as a mediator, which in addition to neuroimaging features, also included the following observed variables: prestroke mRS (known to be strongly correlated with traditional frailty measures like the Rockwood frailty index^34^), diabetes, smoking, hypertension, hypercholesterolemia, history of congestive heart failure, any other cardiac disease, atrial fibrillation, peripheral vascular disease, any other vascular risk factors, preexisting kidney failure, preexisting cancer, prior stroke, prior traumatic brain injury, and any prior major surgery.

Figure 2 shows a causal hypothesis diagram for each proposed model, in which pathway A runs from the independent variable to the mediator, pathway B runs from the mediator to the dependent variable, and pathway C runs directly from the independent variable to the dependent variable. The direct effect is defined as pathway C, and the indirect effect as pathway A × B. The β coefficients for each pathway in the causal hypothesis model as well as β coefficients of the direct effect (pathway C), the indirect effect (pathway A × B), and the total effect (pathway C + [A × B ]) were calculated, along with 95% CIs. Last, we calculated the exact proportion of the effect of the mediator (ie, indirect effect) on the total effect by dividing the indirect effect by the total effect. Furthermore, we used the Pearson χ^2^ test to test goodness of fit^35^ of the data included in each model.

As additional sensitivity analyses, we created the latent variable for the first model (ie, neuroimaging frailty) by including only patients who had available and interpretable 24-hour MRI, adding 3 MRI-specific imaging features (ie, enlarged perivascular spaces score, number of microbleeds, and cortical superficial siderosis). All 2-tailed *P* values ≤.05 were considered significant. Analyses were performed using Stata/MP, version 16.1 (StataCorp LLC).

## Results

Among 1105 patients enrolled in the ESCAPE-NA1 trial, 1102 (99.7%) had an interpretable baseline NCCT for assessing brain frailty measures. The median age was 71 years (IQR, 61-80 years), 548 (49.7%) were female, 554 (50.3%) were male, and 549 (49.8%) were treated with IV nerinetide. Compared with patients 71 years or younger, patients older than 71 years were more often female (310 of 547 [56.7%] vs 238 of 555 [42.9%]; *P* < .001), less often active smokers (34 of 547 [6.2%] vs 198 of 555 [35.7%]; *P* < .001), more often had higher total Fazekas scores of 2 (148 of 547 [27.1%] vs 63 of 555 [11.4%]) and 3-6 (147 of 547 [26.9%] vs 48 of 555 [8.6%]) (*P* < .001), and had lower final infarct volumes (median 5 mL [IQR, 22-99 mL] vs median 27 mL [IQR, 9-86 mL]; *P* = .04). In addition, the older patient group showed similar early ischemic changes (ASPECTS), similar occlusion locations, and similar collateral grades. Among those in the younger group, 487 (87.7%) had a premorbid mRS score of 0 (Table 1).

**Table 1.** Baseline Characteristics

Characteristic	Participants, No. (%) (N = 1102)		*P* value
	≤71 y (n = 555)	>71 y (n = 547)	
Sex			
Female	238 (42.9)	310 (56.7)	<.001
Male	317 (57.1)	237 (43.3)	
Prestroke mRS score^a^			
0	487 (87.7)	407 (74.4)	<.001
1	51 (9.2)	85 (15.7)	
2	16 (2.9)	50 (9.1)	
3	0	3 (1.0)	
Baseline NIHSS score, median (IQR)^b^	17 (12-21)	17 (13-21)	.06
Comorbidities			
Current smoker	198 (35.7)	34 (6.2)	<.001
Peripheral vascular disease	26 (4.7)	33 (6.1)	.30
Hypertension	321 (57.8)	451 (82.4)	<.001
Hyperlipidemia	221 (39.8)	293 (53.6)	<.001
Diabetes			
Type 1	4 (1.0)	4 (1.0)	.01
Type 2	86 (15.5)	123 (22.5)	
Atrial fibrillation	122 (22.0)	265 (48.4)	<.001
Baseline ASPECTS, median (IQR)^c^	8 (7-8)	8 (7-9)	<.001
Total Fazekas score^d^			
0-1	444 (80.0)	252 (46.1)	<.001
2	63 (11.4)	148 (27.1)	
3-6	48 (8.6)	147 (26.9)	
Global cortical atrophy score^e^			
0	518 (93.3)	300 (54.8)	<.001
1	34 (6.1)	186 (34.0)	
2 or 3	3 (1.0)	61 (11.2)	
CC/IT ratio, median (IQR)	0.10 (0.09-0.13)	0.14 (0.12-0.17)	<.001
Lacunes present	97 (17.5)	175 (32.0)	<.001
≥1 Chronic infarction	57 (10.3)	72 (13.2)	.14
Final infarction volume, median (IQR), mL^f^	27 (9-86)	5 (22-99)	.04
Collaterals			
Good	101 (18.2)	89 (16.3)	.52
Moderate	419 (75.5)	433 (79.2)	
Poor	26 (4.7)	22 (4.0)	
Intracranial occlusion location			
ICA	134 (24.1)	121 (22.1)	.41
M1 branch of middle cerebral artery	395 (71.2)	404 (73.9)	
Intravenous nerinetide treatment	265 (47.7)	284 (51.9)	.17
Intravenous alteplase treatment	345 (62.2)	312 (57.0)	.08

Abbreviations: ASPECTS, Alberta Stroke Program Early Computed Tomography Score; CC/IT, intercaudate distance to inner table width; ICA, internal carotid artery; mRS, modified Rankin Scale; NIHSS, National Institutes of Health Stroke Scale.

^a^ Scores range from 0 to 6, with 0 indicating perfect health without symptoms and 6 indicating death.

^b^ Scores range from 0 to 42, with higher scores indicating severe stroke.

^c^ Scores range from 0 to 10, with lower scores indicating more middle cerebral artery regions involved.

^d^ This scale evaluates the degree of white matter disease (WMD) in both periventricular and deep locations, with the score for each location ranging from 0 (no WMD) to 3 (large confluent areas or hypodensities extending to the cortex), combined to generate a total WMD-burden score.

^e^ This pragmatic, qualitative scale evaluates the degree of cortical atrophy by assessing the width of the sulci and the volume of the gyri, ranging from 0 for no atrophy to 3 for severe atrophy.

^f^ Volumes are rounded to whole numbers.

### Model 1, Neuroimaging Measures of Frailty

**Figure 3.**
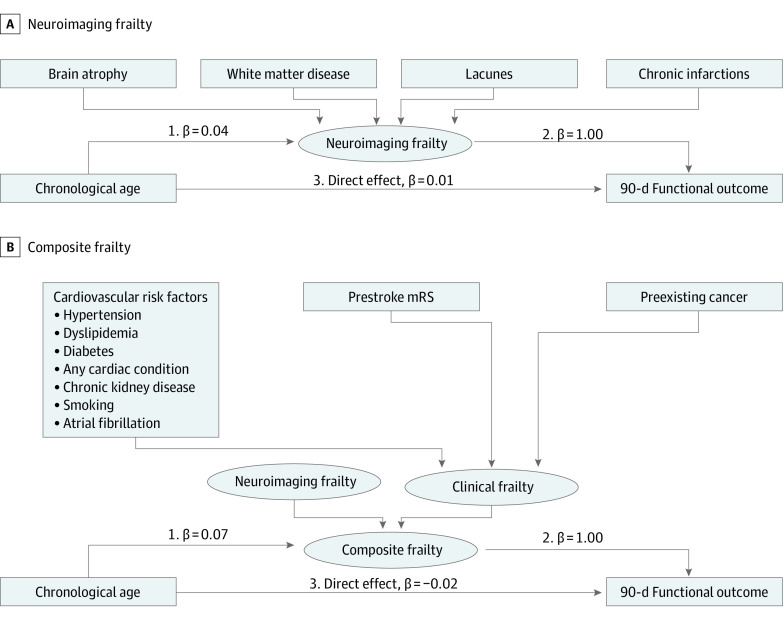
Structural Equation Models Including Different Latent Variables as Possible Mediator Each causal hypothesis diagram includes a latent variable (brain frailty) as a possible mediator for the association of chronological age and 90-day functional outcome. Each model includes a different latent variable: imaging measures of brain frailty (A) and a total construct of brain frailty (B). Adjustments for all models were made for the administration of nerinetide and/or alteplase, onset-to-puncture time, National Institutes of Health Stroke Scale at baseline, and Alberta Stroke Program Early Computed Tomography Score at baseline. Rectangles represent observed variables; ovals represent latent variables. Linear equations are built by using arrows between the variables; 90-day functional outcome was included as an ordinal variable following the full categorical scale (ie, modified Rankin Scale [mRS] scale, which ranges from 0 to 6, with 0 indicating perfect health without symptoms and 6 indicating death).

In model 1 (ie, neuroimaging frailty), we observed a positive association between age and 90-day outcome (including both direct and indirect pathways; note that higher mRS scores indicate worse outcome). For the total effect of age on 90-day outcome, we found that for every 10-year increase in age, there was a 0.47 percentage point increase in the mean mRS (β coefficient of 0.05 per year [95% CI, 0.04-0.06]; *P* < .001). The direct effect of age on 90-day outcome in this model was smaller and nonsignificant: for every 10-year increase in age, there was an average increase of 0.07 percentage points in the mean mRS (β coefficient of 0.01 per year [95% CI, −0.01 to 0.03]; *P* = .49). The indirect effect of age, on the other hand, through the mediator (neuroimaging frailty), showed a significant association: for every 10-year increase in age, there was a 0.4 percentage point increase in the mean mRS (β coefficient of 0.04 per year [95% CI, 0.02-0.06 per year]; *P* < .001). The proportion of the mediation of the indirect effect on the total effect (ie, the mediation of the association between age and 90-day outcome by imaging measures of brain frailty) was 85.1% (0.04 of 0.05; Figure 3A and Table 2). Additional sensitivity analyses (eFigure 2 and eTable 2 in Supplement 1) showed similar results when including only patients with available 24-hour MRI. All models had satisfactory goodness-of-fit metrics.

**Table 2.** Effect Estimates for Each Pathway in the Mediation Models^a^

Frailty model	β Coefficient (95% CI)	*P* value
**Neuroimaging**		
Direct effect	0.01 (−0.01 to 0.03)	.49
Indirect effect	0.04 (0.02 to 0.06)	<.001
Total effect	0.05 (0.04 to 0.06)	<.001
Relative proportion of indirect effect, %^b^	85.1	NA
**Composite**		
Direct effect	−0.02 (−0.01 to 0.03)	.26
Indirect effect	0.07 (0.03 to 0.10)	.001
Total effect	0.05 (0.04 to 0.06)	<.001
Relative proportion of indirect effect, %^b^	100	NA

Abbreviation: NA, not applicable.

^a^ Effect estimates are derived from linear regression models and are adjusted for baseline Alberta Stroke Program Early Computed Tomography Score, National Institutes of Health Stroke Scale, onset-to-puncture time, nerinetide treatment, and alteplase treatment.

^b^ Relative proportions of the indirect effect were calculated by dividing the absolute effect size of the indirect effect by the absolute effect size of the total effect of age on 90-day outcome. Because the proportion cannot exceed 100%, the composite frailty model is essentially 100%.

### Model 2, Composite Frailty—Neuroimaging and Clinical Features Associated With Frailty

In model 2, the total effect of age on 90-day outcome showed that for every 10-year increase, there was a 0.5 percentage point increase in the mean mRS (β coefficient = 0.05 [95% CI, 0.04-0.06]; *P* < .001). The direct effect was not significant (β coefficient, −0.02 [95% CI, −0.01 to 0.03]; *P* = .26). The indirect effect, on the other hand, was larger and significant: for every 10-year increase in age, there was a 0.7 percentage point increase in the mean mRS (β coefficient, 0.07 per year [95% CI, 0.03-0.10]; *P* = .001); thus, the total brain-frailty construct was essentially 100% of the total effect (relative proportions of the indirect effect were calculated by dividing the absolute effect size of the indirect effect by the absolute effect size of the total effect of age on 90-day outcome; because the proportion cannot exceed 100%, the composite frailty model is essentially 100%) (Figure 3B and Table 2). Additional sensitivity analyses (eFigure 2 and eTable 2 in Supplement 1) showed similar results when including only patients with available 24-hour MRI.

## Discussion

In this post hoc cohort study of 1102 patients with AIS undergoing EVT, we found that the association of age with the 90-day mRS was mostly mediated by neuroimaging measures of frailty determined pragmatically on NCCT (ie, 85.1% of the total effect). When combining both imaging measures and clinical variables to create composite frailty as an aggregate mediator, this essentially contributed to the entirety of the total association of age with 90-day mRS.

Neuroimaging frailty is not routinely considered in the evaluation of patients with injuries like AIS in current practice nor is it considered in patient selection or randomization for randomized clinical trials. Recent studies that explored the association of brain frailty measures with functional outcome after EVT for patients with AIS only focused on individual features, often considering only a single measure like WMD or atrophy, using conventional analytical approaches like logistic regression, adjusted for age and other baseline measures.^15,16,18^ However, with such conventional approaches, it is difficult to meaningfully quantify the association of brain frailty with age-related differences in poststroke outcomes. Structural equation modeling is an attractive solution, as it allows us to create a latent variable estimated from several measurable variables as a combined construct as well as to create a hypothetical causal diagram in which this latent variable is considered as a possible mediator. To our knowledge, there has been 1 retrospective study that used SEM to build a theoretical construct of several measurable features in the exploration of predicting poststroke outcomes.^36^ The study included 453 patients with AIS and created a latent variable, or effective reserve, which was defined by age, systolic blood pressure, and intracranial volume (just a single brain-frailty measure, complexly determined using volumetric software), and found that a prediction model for the 90-day mRS that included effective reserve was associated with better performances compared with a model without this construct (eg, only including measurable variables). The study also showed that a higher effective reserve was associated with more favorable functional poststroke outcomes. In the current study, we added to the prior literature by combining several pragmatic, easy-to-use scales that assessed key measures on neuroimaging of frailty, including cortical atrophy, intercaudate distance (a measure of subcortical atrophy), WMD, lacunes, and chronic infarctions on routinely acquired NCCT, into an aggregate neuroimaging frailty construct. Such measures are more feasible to implement in emergency situations such as acute stroke diagnostics as opposed to sophisticated volumetric features. Through our SEM-based mediation analyses, we were able to quantify the remarkable extent to which brain frailty mediated the association between age and 90-day functional outcome.

This finding has important clinical implications. If most of the association of chronological age was mediated by brain frailty measures, then this further underscores the importance of not using chronological age alone to make fatalistic outcome predictions or to exclude patients from therapy. Consideration of neuroimaging features of frailty, such as atrophy or small vessel disease (eg, lacunes, WMD), may help health care teams working with patients who have had a stroke make a more nuanced prognostication for their patients. Similar considerations may apply to other acquired brain injuries like intracerebral hemorrhage, traumatic brain injury,^22^ and demyelinating disorders. Our results should also motivate researchers who are trying to build and improve prediction models after EVT (eg, MR PREDICTS^37^) to incorporate such measures in their models.

Whereas chronological age is typically considered a nonmodifiable predictor of 90-day outcome, we found that neuroimaging frailty mediated the majority of the effect of age on 90-day outcomes. Aspects of brain frailty could to some extent^38^ be modifiable or preventable by managing cardiovascular risk factors (eg, diabetes, hypertension, and dyslipidemia to mitigate chronic infarct burden).

### Limitations

This study has some limitations. First, all participants enrolled in the ESCAPE-NA1 trial needed favorable baseline imaging and premorbid functional status. Over 80% had premorbid mRS of 0, since the frailest patients were probably excluded from enrollment. This made us likely to underestimate the extent of mediation by clinical frailty measures. In the general population, clinical and neuroimaging-derived brain frailty would be expected to have an even higher impact. This may have limited the generalizability of our results to the broader population undergoing EVT, given, for example, the recent expansion of EVT indications to include those patients who have had a stroke with more extensive ischemic changes.^39,40^ Nonetheless, we think that this would not have altered the message of the study. Furthermore, prestroke cognitive concerns were not routinely captured in this study. However, we hypothesize that patients with a greater burden of neuroimaging frailty markers may be at greater risk to develop prestroke cognitive issues, which in turn may affect their poststroke recovery (irrespective of age). Future studies should consider incorporating measures of assessing prestroke cognitive functioning.^41^ Second, all included participants underwent EVT, preventing estimations of treatment effect sizes of EVT vs medical management in patients with low or high brain frailty. Third, the applied atrophy scales were originally developed for evaluation of neurodegenerative disorders on MRI in a nonemergency setting. However, such scales have since been successfully adapted for CT-based analyses.^15^ In addition, we performed sensitivity analyses including only those patients with available and interpretable MRI and achieved similar results. Nevertheless, these imaging features did not include somatic parameters such as sarcopenia, which may also influence frailty. Fourthly, global clinical frailty indexes were not evaluated as part of the ESCAPE-NA1 trial, which might have achieved stronger mediation of the age and mRS association. However, we included several relevant clinical variables associated with frailty, including prestroke mRS, which has shown an especially strong correlation.^34^

## Conclusions

In this cohort study analyzing the ESCAPE-NA1 randomized clinical trial, brain frailty mediated the association of age and 90-day outcome after EVT, with most of the effect mediated by neuroimaging features. This work underscores the importance of considering brain frailty, as opposed to chronological age alone, in predicting poststroke outcomes. These results suggest that neuroimaging-derived brain frailty should be included in future randomized clinical trials, whether as an adjusting variable in final analyses or as a means to stratify for randomization. Further studies are needed to understand whether mitigating brain frailty or, conversely, increasing brain reserve can translate into improved poststroke outcomes among older patients, and future work should focus on creating a clinically usable aggregate imaging frailty score.

## Notes

Supplement 1. **eFigure 1.** Measurement of the Hemi-Intercaudate Distance (h-ICD) and Inner Table Width (ITW) **eFigure 2.** Structural Equation Models Including Different Latent Variables as Possible Mediator, MRI Only (n = 568) **eTable 1.** Imaging Core Laboratory Members **eTable 2.** Effect Estimates for Each Pathway in the Mediation Models, MRI Only (N = 568)

Supplement 2. **Nonauthor Collaborators.** ESCAPE-NA1 investigators

Supplement 3.
Data Sharing Statement
