# Supplementary material for: Mediation of Age and Thrombectomy Outcome by Neuroimaging Markers of Frailty in Patients With Stroke
Source: JAMA Netw Open. 2024 Jan 2;7(1):e2349628. doi: 10.1001/jamanetworkopen.2023.49628 (PMC10762575; doi:10.1001/jamanetworkopen.2023.49628)
Supplement: Supplement 1. — eFigure 1. Measurement of the Hemi-Intercaudate Distance (h-ICD) and Inner Table Width (ITW) eFigure 2. Structural Equation Models Including Different Latent Variables as Possible Mediator, MRI Only (n = 568) eTable 1. Imaging Core Laboratory Members eTable 2. Effect Estimates for Each Pathway in the Mediation Models, MRI Only (N = 568) [file jamanetwopen-e2349628-s001.pdf]

Supplemental Online Content

Benali F, Singh N, Fladt J, et al. Mediation of age and thrombectomy outcome by neuroimaging markers of frailty in patients with stroke. JAMA Netw Open. 2023;6(12):e2349628. doi:10.1001/jamanetworkopen.2023.49628

eFigure 1. Measurement of the Hemi-Intercaudate Distance (h-ICD) and Inner Table Width (ITW)
eFigure 2. Structural Equation Models Including Different Latent Variables as Possible Mediator, MRI Only (n = 568)
eTable 1. Imaging Core Laboratory Members
eTable 2. Effect Estimates for Each Pathway in the Mediation Models, MRI Only (N = 568)

This supplemental material has been provided by the authors to give readers additional information about their work.

© 2024 Benali F et al. *JAMA Network Open*.

eFigure 1. Measurement of the Hemi-Intercaudate Distance (h-ICD) and Inner Table Width (ITW)

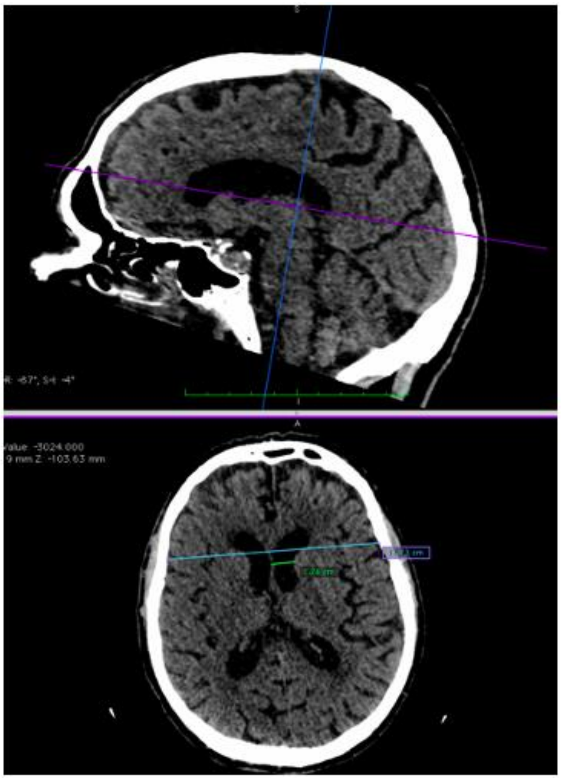

eFigure 1. Non-Contrast CT (NCCT) is shown in the sagittal plane (top) and axial plane (bottom). Measurements of the h-ICD are performed on the contralateral side to the AIS to account for any ischemic areas or hemorrhages associated with potential edema. To determine the h-ICD, the distance in mm between the septum pellucidum and the caudate head is measured (green line). In a second step, the h-ICD is multiplied by 2 to obtain the ICD. The ITW in mm is measured on the same sectional plane (turquoise line). Measurements are done with multiplanar reconstruction (MPR), to assure an accurate anterior-posterior commissural (APC) line (purple line). Finally, the intercaudate distance to inner table width ratio (CC/IT) is automatically calculated.

© 2024 Benali F et al. *JAMA Network Open*.

eFigure 2. Structural Equation Models Including Different Latent Variables As Possible Mediator, MRI Only (n = 568)

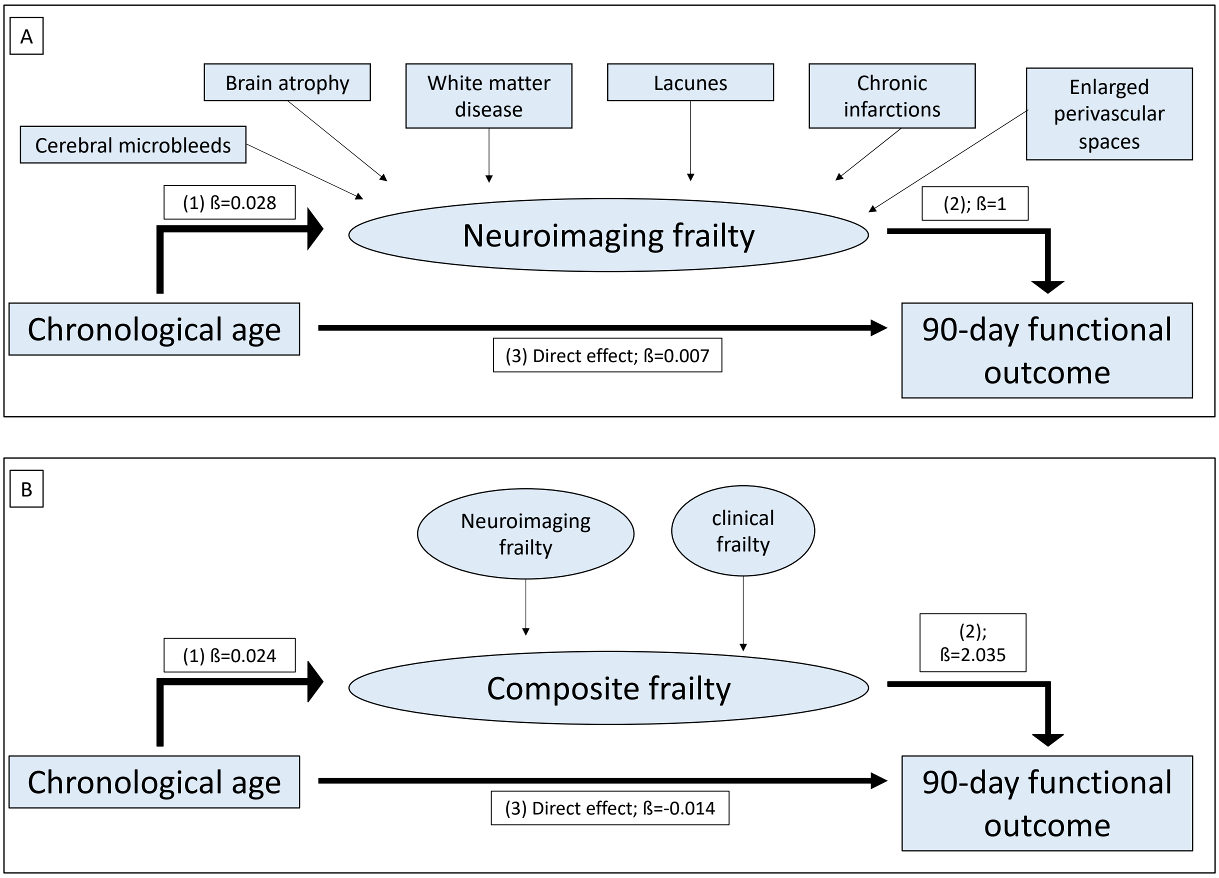

eFigure 2. Shown are 2 causal diagrams including a latent variable (brain-frailty) as possible mediator for the association of chronological age and 90-day functional outcome. Each model includes a different latent variable: (A) shows imaging-based brain frailty, (B) shows a total construct of brain frailty. Adjustments for all models were made for the administration of Nerinetide, Alteplase, Onset to puncture time, NIHSS at baseline and ASPECTS at baseline.

© 2024 Benali F et al. *JAMA Network Open*.

eTable 1. Imaging Core Laboratory Members

Name	Subspecialty	Stroke imaging experience, years	Role in the core lab
Faysal Benali	Neuroradiology (diagnostic)	4	Cortical atrophy, subcortical atrophy and WMD burden assessments
Johanna Ospel	General radiology	5	FIV delineations
Fouzi Bala	Neuroradiology (diagnostic & interventional)	7	Cortical atrophy, subcortical atrophy and WMD burden assessments
Joachim Fladt	Stroke neurology	7	Cortical atrophy, subcortical atrophy and WMD burden assessments
Nishita Singh	Stroke neurology	7	Cortical atrophy, subcortical atrophy and WMD burden assessments
Aravind Ganesh	Stroke neurology	10	Cortical atrophy, subcortical atrophy and WMD burden assessments *

* In case of $\geq$ 2 grade disagreements.

© 2024 Benali F et al. *JAMA Network Open*.

eTable 2. Effect Estimates for Each Pathway in the Mediation Models, MRI Only (N = 568)

**Neuroimaging frailty**	ß-coefficient	P-value	95% CI
Direct effect	0.007	0.59	-0.017; 0.030
Indirect effect	**0.028**	**0.01**	**0.007-0.050**
Total effect	**0.035**	**<0.001**	**0.025-0.045**
Relative proportion of indirect effect		**80%**	
**Composite frailty**	ß-coefficient	P-value	95% CI
Direct effect	-0.014	0.34	-0.042; 0.015
Indirect effect	**0.049**	**<0.001**	**0.022-0.076**
Total effect	**0.035**	**<0.001**	**0.025-0.045**
Relative proportion of indirect effect		**~100%**	

© 2024 Benali F et al. *JAMA Network Open*.
