# Supplementary material for: Mediation of Age and Thrombectomy Outcome by Neuroimaging Markers of Frailty in Patients With Stroke
Source: JAMA Netw Open. 2024 Jan 2;7(1):e2349628. doi: 10.1001/jamanetworkopen.2023.49628 (PMC10762575; doi:10.1001/jamanetworkopen.2023.49628)
Supplement: Supplement 2. — Nonauthor Collaborators. ESCAPE-NA1 investigators [file jamanetwopen-e2349628-s002.pdf]

Supplemental Online Content: Nonauthor Collaborators

*Indicates required information. Only first name, last name, and suffix will appear in PubMed.

*Group Name(s): ESCAPE-NA1 Investigators							
***First Name and Middle Initial(s)**	***Last Name**	***Suffix (eg, Jr, III)**	Academic Degrees	Institution	Location (city, state/province, country)	Role or Contribution, eg, chair, principal investigator	Group (if more than 1 Group listed in the byline) and/or Subgroup (eg, Steering Committee)
Philip A.	Barber		MD	Foothills Medical Centre, University of Calgary	Calgary, AB, Canada	Collaborator	
Eric E.	Smith		MD	Foothills Medical Centre, University of Calgary	Calgary, AB, Canada	Collaborator	
Simerpreet	Bal		MD	Foothills Medical Centre, University of Calgary	Calgary, AB, Canada	Collaborator	
Suresh	Subramanian		MD	Foothills Medical Centre, University of Calgary	Calgary, AB, Canada	Collaborator	
Steven	Peters		MD	Foothills Medical Centre, University of Calgary	Calgary, AB, Canada	Collaborator	
Philippe	Couillard		MD	Foothills Medical Centre, University of Calgary	Calgary, AB, Canada	Collaborator	
Gary	Klein		MD	Foothills Medical Centre, University of Calgary	Calgary, AB, Canada	Collaborator	
Peter	Stys		MD	Foothills Medical Centre, University of Calgary	Calgary, AB, Canada	Collaborator	
Shelagh B.	Coutts		MD	Foothills Medical Centre, University of Calgary	Calgary, AB, Canada	Collaborator	
John	Wong		MD	Foothills Medical Centre, University of Calgary	Calgary, AB, Canada	Collaborator	
Alim	Mitha		MD	Foothills Medical Centre, University of Calgary	Calgary, AB, Canada	Collaborator	
Muneer	Eesa		MD	Foothills Medical Centre, University of Calgary	Calgary, AB, Canada	Collaborator	
William	Morrish		MD	Foothills Medical Centre, University of Calgary	Calgary, AB, Canada	Collaborator	
Saad	Alqatani		MD	Foothills Medical Centre, University of Calgary	Calgary, AB, Canada	Collaborator	
Nima	Kashani		MD	Foothills Medical Centre, University of Calgary	Calgary, AB, Canada	Collaborator	
Prasanna	Venkatesan		MD	Foothills Medical Centre, University of Calgary	Calgary, AB, Canada	Collaborator	
Ericka	Teleg		MD	Foothills Medical Centre, University of Calgary	Calgary, AB, Canada	Collaborator	

*Indicates required information. Only first name, last name, and suffix will appear in PubMed.

*First Name and Middle Initial(s)	*Last Name	*Suffix (eg, Jr, III)	Academic Degrees	Institution	Location (city, state/province, country)	Role or Contribution, eg, chair, principal investigator	Group (if more than 1 Group listed in the byline) and/or Subgroup (eg, Steering Committee)
Amith	Sitaram		MD	Foothills Medical Centre, University of Calgary	Calgary, AB, Canada	Collaborator	
Brett	Graham		MD	Foothills Medical Centre, University of Calgary	Calgary, AB, Canada	Collaborator	
Stephen van	Gaal		MD	Foothills Medical Centre, University of Calgary	Calgary, AB, Canada	Collaborator	
Aimen	Moussaddy		MD	Foothills Medical Centre, University of Calgary	Calgary, AB, Canada	Collaborator	
Debabrata	Chakraborty		MD	Foothills Medical Centre, University of Calgary	Calgary, AB, Canada	Collaborator	
Nicholar	Maraj		MD	Foothills Medical Centre, University of Calgary	Calgary, AB, Canada	Collaborator	
Andrew	Lockey		MD	Foothills Medical Centre, University of Calgary	Calgary, AB, Canada	Collaborator	
Shuo	Chen		MD	Foothills Medical Centre, University of Calgary	Calgary, AB, Canada	Collaborator	
Ravinder	Singh		MD	Foothills Medical Centre, University of Calgary	Calgary, AB, Canada	Collaborator	
Abdulaziz Sulaiman	Alsultan		MD	Foothills Medical Centre, University of Calgary	Calgary, AB, Canada	Collaborator	
Ria	Asunsian		MD	Foothills Medical Centre, University of Calgary	Calgary, AB, Canada	Collaborator	
Dominic	Tse		MD	Foothills Medical Centre, University of Calgary	Calgary, AB, Canada	Collaborator	
Darshan	Doshi		MD	Foothills Medical Centre, University of Calgary	Calgary, AB, Canada	Collaborator	
Ondrej	Volny		MD	Foothills Medical Centre, University of Calgary	Calgary, AB, Canada	Collaborator	
Piyush	Ojha		MD	Foothills Medical Centre, University of Calgary	Calgary, AB, Canada	Collaborator	
Ankur	Wadhwa		MD	Foothills Medical Centre, University of Calgary	Calgary, AB, Canada	Collaborator	
Martha	Marko		MD	Foothills Medical Centre, University of Calgary	Calgary, AB, Canada	Collaborator	
Nishita	Singh		MD	Foothills Medical Centre, University of Calgary	Calgary, AB, Canada	Collaborator	

Supplemental Online Content: Nonauthor Collaborators

*Indicates required information. Only first name, last name, and suffix will appear in PubMed.

***First Name and Middle Initial(s)**	***Last Name**	***Suffix (eg, Jr, III)**	Academic Degrees	Institution	Location (city, state/province, country)	Role or Contribution, eg, chair, principal investigator	Group (if more than 1 Group listed in the byline) and/or Subgroup (eg, Steering Committee)
Sanchea	Wasyliw		MD	Foothills Medical Centre, University of Calgary	Calgary, AB, Canada	Collaborator	
Karla J.	Ryckborst		RN	Foothills Medical Centre, University of Calgary	Calgary, AB, Canada	Collaborator	
Carol	Kenney		RN	Foothills Medical Centre, University of Calgary	Calgary, AB, Canada	Collaborator	
Supriya	Save			Foothills Medical Centre, University of Calgary	Calgary, AB, Canada	Collaborator	
Anitha	Jambula			Foothills Medical Centre, University of Calgary	Calgary, AB, Canada	Collaborator	
Nancy	Newcommon			Foothills Medical Centre, University of Calgary	Calgary, AB, Canada	Collaborator	
Gavin	Hull			Foothills Medical Centre, University of Calgary	Calgary, AB, Canada	Collaborator	
Darcy	Blackstock			Foothills Medical Centre, University of Calgary	Calgary, AB, Canada	Collaborator	
Sharon	Kiszczak			Foothills Medical Centre, University of Calgary	Calgary, AB, Canada	Collaborator	
Leslie	Zimmel			Foothills Medical Centre, University of Calgary	Calgary, AB, Canada	Collaborator	
Michelle	Wright			Foothills Medical Centre, University of Calgary	Calgary, AB, Canada	Collaborator	
Cari	Jahraus			Foothills Medical Centre, University of Calgary	Calgary, AB, Canada	Collaborator	
Linda	Andersen			Foothills Medical Centre, University of Calgary	Calgary, AB, Canada	Collaborator	
Shelly	Bohn			Foothills Medical Centre, University of Calgary	Calgary, AB, Canada	Collaborator	
Joseph	Paul			Foothills Medical Centre, University of Calgary	Calgary, AB, Canada	Collaborator	
Brian H.	Buck		MD	University of Alberta Hospital	Edmonton, AB, Canada	Site PI	
Kenneth S.	Butcher		MD	University of Alberta Hospital	Edmonton, AB, Canada	Site PI	

Supplemental Online Content: Nonauthor Collaborators

*Indicates required information. Only first name, last name, and suffix will appear in PubMed.

***First Name and Middle Initial(s)**	***Last Name**	***Suffix (eg, Jr, III)**	Academic Degrees	Institution	Location (city, state/province, country)	Role or Contribution, eg, chair, principal investigator	Group (if more than 1 Group listed in the byline) and/or Subgroup (eg, Steering Committee)
Ashfaq	Shuaib		MD	University of Alberta Hospital	Edmonton, AB, Canada	Collaborator	
Tom	Jeerakathil		MD	University of Alberta Hospital	Edmonton, AB, Canada	Collaborator	
Glen	Jickling		MD	University of Alberta Hospital	Edmonton, AB, Canada	Collaborator	
Derek J.	Emery		MD	University of Alberta Hospital	Edmonton, AB, Canada	Collaborator	
Jeremy	Rempel		MD	University of Alberta Hospital	Edmonton, AB, Canada	Collaborator	
Richard J.	Ower		MD	University of Alberta Hospital	Edmonton, AB, Canada	Collaborator	
Robert	Ashforth		MD	University of Alberta Hospital	Edmonton, AB, Canada	Collaborator	
Tom	Yeo		MD	University of Alberta Hospital	Edmonton, AB, Canada	Collaborator	
Trevor B.	Kotylak		MD	University of Alberta Hospital	Edmonton, AB, Canada	Collaborator	
Cian	O;Kelly		MD	University of Alberta Hospital	Edmonton, AB, Canada	Collaborator	
Michael	Chow		MD	University of Alberta Hospital	Edmonton, AB, Canada	Collaborator	
Mizaffar	Siddiqui		MD	University of Alberta Hospital	Edmonton, AB, Canada	Collaborator	
Maher	Saqqur		MD	University of Alberta Hospital	Edmonton, AB, Canada	Collaborator	
Atlantic	D'Souza		MD	University of Alberta Hospital	Edmonton, AB, Canada	Collaborator	
Mar	Lloret		MD	University of Alberta Hospital	Edmonton, AB, Canada	Collaborator	
Asif	Butt		MD	University of Alberta Hospital	Edmonton, AB, Canada	Collaborator	
Ali	Nomani		MD	University of Alberta Hospital	Edmonton, AB, Canada	Collaborator	
Hayrapet	Kalashyan		MD	University of Alberta Hospital	Edmonton, AB, Canada	Collaborator	

Supplemental Online Content: Nonauthor Collaborators

*Indicates required information. Only first name, last name, and suffix will appear in PubMed.

*First Name and Middle Initial(s)	*Last Name	*Suffix (eg, Jr, III)	Academic Degrees	Institution	Location (city, state/province, country)	Role or Contribution, eg, chair, principal investigator	Group (if more than 1 Group listed in the byline) and/or Subgroup (eg, Steering Committee)
Sibi	Thirunavukkarasu		MD	University of Alberta Hospital	Edmonton, AB, Canada	Collaborator	
Juline	Jabs		RN	University of Alberta Hospital	Edmonton, AB, Canada	Collaborator	
Paige	Fairall			University of Alberta Hospital	Edmonton, AB, Canada	Collaborator	
Lori	Piquette		RN	University of Alberta Hospital	Edmonton, AB, Canada	Collaborator	
Stephen J.	Phillips		MBBS FRCPC	Queen Elizabeth II Health Science Centre, Dalhousie University	Halifax, NS, Canada	Site PI	
A. Laine	Green		MD, FRCPC	Queen Elizabeth II Health Science Centre, Dalhousie University	Halifax, NS, Canada	Collaborator	
Gordon J.	Gubitz		MD, FRCPC	Queen Elizabeth II Health Science Centre, Dalhousie University	Halifax, NS, Canada	Collaborator	
Jens O.	Heidenreich		MD	Queen Elizabeth II Health Science Centre, Dalhousie University	Halifax, NS, Canada	Collaborator	
Thien	Huynh		MD, FRCPC	Queen Elizabeth II Health Science Centre, Dalhousie University	Halifax, NS, Canada	Collaborator	
Jai J. S.	Shankar		MD, FRCPC	Queen Elizabeth II Health Science Centre, Dalhousie University	Halifax, NS, Canada	Collaborator	
William J.	Maloney		MD, FRCPC	Queen Elizabeth II Health Science Centre, Dalhousie University	Halifax, NS, Canada	Collaborator	
Robert	Vandorpe		MD, FRCPC	Queen Elizabeth II Health Science Centre, Dalhousie University	Halifax, NS, Canada	Collaborator	
Matthias H.	Schmidt		MD, FRCPC	Queen Elizabeth II Health Science Centre, Dalhousie University	Halifax, NS, Canada	Collaborator	
Gwynedd E.	Pickett		MD, FRCSC	Queen Elizabeth II Health Science Centre, Dalhousie University	Halifax, NS, Canada	Collaborator	
Adrienne	Weeks		MD, PhD, FRCSC	Queen Elizabeth II Health Science Centre, Dalhousie University	Halifax, NS, Canada	Collaborator	
Juditch	Jarrett		RN, CCRP	Queen Elizabeth II Health Science Centre, Dalhousie University	Halifax, NS, Canada	Collaborator	
Debbie	MacDonald		RN, CCRP	Queen Elizabeth II Health Science Centre, Dalhousie University	Halifax, NS, Canada	Collaborator	

Supplemental Online Content: Nonauthor Collaborators

*Indicates required information. Only first name, last name, and suffix will appear in PubMed.

*First Name and Middle Initial(s)	*Last Name	*Suffix (eg, Jr, III)	Academic Degrees	Institution	Location (city, state/province, country)	Role or Contribution, eg, chair, principal investigator	Group (if more than 1 Group listed in the byline) and/or Subgroup (eg, Steering Committee)
Joanna	Arsenault		CRP	Queen Elizabeth II Health Science Centre, Dalhousie University	Halifax, NS, Canada	Collaborator	
Ruth	Kinnear		RN	Queen Elizabeth II Health Science Centre, Dalhousie University	Halifax, NS, Canada	Collaborator	
Jennifer L.	Mandzia		MD, PhD	London Health Science Centre, Western Unive	London, ON, Canada	Site PI	
Michael	Mayich		MD	London Health Science Centre, Western Unive	London, ON, Canada	Site PI	
Melfort	Boulton		MD, PhD	London Health Science Centre, Western Unive	London, ON, Canada	Collaborator	
Maria Bres	Bullrich		MD	London Health Science Centre, Western Unive	London, ON, Canada	Collaborator	
Sebastian	Fridman		MD	London Health Science Centre, Western Unive	London, ON, Canada	Collaborator	
Ruba	Kiwan		MD	London Health Science Centre, Western Unive	London, ON, Canada	Collaborator	
Donald	Lee		MD	London Health Science Centre, Western Unive	London, ON, Canada	Collaborator	
Stephen	Lownie		MD	London Health Science Centre, Western Unive	London, ON, Canada	Collaborator	
Alexander V.	Khaw		MD	London Health Science Centre, Western Unive	London, ON, Canada	Collaborator	
Sachin K.	Pandey		MD	London Health Science Centre, Western Unive	London, ON, Canada	Collaborator	
Manas	Sharma		MD	London Health Science Centre, Western Unive	London, ON, Canada	Collaborator	
Luciano A.	Sposato		MD	London Health Science Centre, Western Unive	London, ON, Canada	Collaborator	
Kevin	Wade		MD	London Health Science Centre, Western Unive	London, ON, Canada	Collaborator	
Beth	Beauchamp		RN	London Health Science Centre, Western Unive	London, ON, Canada	Collaborator	
Lindsay	Lambourn		RN	London Health Science Centre, Western Unive	London, ON, Canada	Collaborator	
Belinda	Amato-Marziali			London Health Science Centre, Western Unive	London, ON, Canada	Collaborator	
Daniel	Roy		MD	CHUM	Montreal, QC, Canada	Site PI	
Nicole	Deneault		MD	CHUM	Montreal, QC, Canada	Collaborator	
Yan	Deschaintre		MD	CHUM	Montreal, QC, Canada	Collaborator	
Laura C.	Gioia		MD	CHUM	Montreal, QC, Canada	Collaborator	
Grégory	Jacquin		MD	CHUM	Montreal, QC, Canada	Collaborator	
Céline	Odier		MD	CHUM	Montreal, QC, Canada	Collaborator	
Christian	Stapf		MD, INR	CHUM	Montreal, QC, Canada	Collaborator	
Daniela	Iancu		MD	CHUM	Montreal, QC, Canada	Collaborator	
Jean	Raymond		MD	CHUM	Montreal, QC, Canada	Collaborator	
Alain	Weill		MD	CHUM	Montreal, QC, Canada	Collaborator	
Marlène	Lapierre		BSc	CHUM	Montreal, QC, Canada	Rearch Nurse Coordinator	
Nadia	Jadil			CHUM	Montreal, QC, Canada	Research administrative assistant	
Judlène	Jolteus		Bsc	CHUM	Montreal, QC, Canada	Clinical Stroke Nurse	

*Indicates required information. Only first name, last name, and suffix will appear in PubMed.

*First Name and Middle Initial(s)	*Last Name	*Suffix (eg, Jr, III)	Academic Degrees	Institution	Location (city, state/province, country)	Role or Contribution, eg, chair, principal investigator	Group (if more than 1 Group listed in the byline) and/or Subgroup (eg, Steering Committee)
Jeanne	Teitelbaum		MD	Montreal Neurological Institute, McGill Univer	Montreal, QC, Canada	Site PI	
Mark	Angle		MD	Montreal Neurological Institute, McGill Univer	Montreal, QC, Canada	Collaborator	
Mathew	Hannouche		MD	Montreal Neurological Institute, McGill Univer	Montreal, QC, Canada	Collaborator	
Mohamed	Badawy		MD	Montreal Neurological Institute, McGill Univer	Montreal, QC, Canada	Collaborator	
Justin	Letourneau		MD	Montreal Neurological Institute, McGill Univer	Montreal, QC, Canada	Collaborator	
Maria	Cortes		MD	Montreal Neurological Institute, McGill Univer	Montreal, QC, Canada	Collaborator	
Grant	Linnell		Md	Montreal Neurological Institute, McGill Univer	Montreal, QC, Canada	Collaborator	
Donatella	Tampieri		MD	Montreal Neurological Institute, McGill Univer	Montreal, QC, Canada	Collaborator	
Lucy	Vieira		MD	Montreal Neurological Institute, McGill Univer	Montreal, QC, Canada	Collaborator	
Aimen	Moussaddy		MD	Montreal Neurological Institute, McGill Univer	Montreal, QC, Canada	Collaborator	
Catherine	Legault		MD	Montreal Neurological Institute, McGill Univer	Montreal, QC, Canada	Collaborator	
Liam	Durcan		MD	Montreal Neurological Institute, McGill Univer	Montreal, QC, Canada	Collaborator	
Angela	Moore		MSc	Montreal Neurological Institute, McGill Univer	Montreal, QC, Canada	Collaborator	
Erin	Cole		MSc	Montreal Neurological Institute, McGill Univer	Montreal, QC, Canada	Collaborator	
Claire	Magnussen		PhD	Montreal Neurological Institute, McGill Univer	Montreal, QC, Canada	Collaborator	
Kristiana	Salmon		EMBA	Montreal Neurological Institute, McGill Univer	Montreal, QC, Canada	Collaborator	
Salma	Khalil		MSc	Montreal Neurological Institute, McGill Univer	Montreal, QC, Canada	Collaborator	
Isabelle	Desloges		MSc	Montreal Neurological Institute, McGill Univer	Montreal, QC, Canada	Collaborator	
Lindsay	Waxman		MSc	Montreal Neurological Institute, McGill Univer	Montreal, QC, Canada	Collaborator	
Wynne	Abdon		BScN	Montreal Neurological Institute, McGill Univer	Montreal, QC, Canada	Collaborator	
Sonia	Lai		BSc	Montreal Neurological Institute, McGill Univer	Montreal, QC, Canada	Collaborator	
Dar	Dowlatshahi		PhD	Ottawa Hospital, University of Ottawa	Ottawa, ON, Canada	Site PI	
Daniela	Iancu		MSc	Ottawa Hospital, University of Ottawa	Ottawa, ON, Canada	Collaborator	
Michel	Shamy		MD	Ottawa Hospital, University of Ottawa	Ottawa, ON, Canada	Collaborator	
Grant	Stotts		MD	Ottawa Hospital, University of Ottawa	Ottawa, ON, Canada	Collaborator	
Dylan	Blacquiere		MD	Ottawa Hospital, University of Ottawa	Ottawa, ON, Canada	Collaborator	
Howard	Lesiuk		MD	Ottawa Hospital, University of Ottawa	Ottawa, ON, Canada	Collaborator	
Aiman	Quateen		MD	Ottawa Hospital, University of Ottawa	Ottawa, ON, Canada	Collaborator	
Brian	Drake		MB	Ottawa Hospital, University of Ottawa	Ottawa, ON, Canada	Collaborator	
Brian	Dewar		MLIS	Ottawa Hospital, University of Ottawa	Ottawa, ON, Canada	Collaborator	
Zeinab	Daham			Ottawa Hospital, University of Ottawa	Ottawa, ON, Canada	Collaborator	
Michael E.	Kelly		MD, PhD	Royal University Hospital, University of Saskat	Saskatoon, SK, Canada	Site PI	
Gary	Hunter		MD, FRCPC	Royal University Hospital, University of Saskat	Saskatoon, SK, Canada	Collaborator	

Supplemental Online Content: Nonauthor Collaborators

*Indicates required information. Only first name, last name, and suffix will appear in PubMed.

*First Name and Middle Initial(s)	*Last Name	*Suffix (eg, Jr, III)	Academic Degrees	Institution	Location (city, state/province, country)	Role or Contribution, eg, chair, principal investigator	Group (if more than 1 Group listed in the byline) and/or Subgroup (eg, Steering Committee)
Lissa M.	Peeling		MD, FRCSC	Royal University Hospital, University of Saskat	Saskatoon, SK, Canada	Collaborator	
Brett R.	Graham		MD, FRCPC	Royal University Hospital, University of Saskat	Saskatoon, SK, Canada	Collaborator	
Ruth	Whelan		BSN, MSN	Royal University Hospital, University of Saskat	Saskatoon, SK, Canada	Collaborator	
Aaron J.	Garner		BSN	Royal University Hospital, University of Saskat	Saskatoon, SK, Canada	Collaborator	
Lilian	Urroz		MD	Royal University Hospital, University of Saskat	Saskatoon, SK, Canada	Collaborator	
Sharleen	Maley		BA	Royal University Hospital, University of Saskat	Saskatoon, SK, Canada	Collaborator	
Frank L.	Silver		MD	University Health Network, University of Toroi	Toronto ON, Canada	Site PI	
Leanne	Casaubon		MD	University Health Network, University of Toroi	Toronto ON, Canada	Collaborator	
Aleksanda	Pikula		MD	University Health Network, University of Toroi	Toronto ON, Canada	Collaborator	
Martin del	Campo		MD	University Health Network, University of Toroi	Toronto ON, Canada	Collaborator	
Joanna	Schaafsma		MD	University Health Network, University of Toroi	Toronto ON, Canada	Collaborator	
Cheryl	Jaigobin		MD	University Health Network, University of Toroi	Toronto ON, Canada	Collaborator	
Timo	Krings		MD	University Health Network, University of Toroi	Toronto ON, Canada	Collaborator	
Vitor	Pereira		MD	University Health Network, University of Toroi	Toronto ON, Canada	Collaborator	
Ronit	Agid		MD	University Health Network, University of Toroi	Toronto ON, Canada	Collaborator	
Richard	Farb		MD	University Health Network, University of Toroi	Toronto ON, Canada	Collaborator	
Libby	Kalman		RN	University Health Network, University of Toroi	Toronto ON, Canada	Collaborator	
Anne	Cayley		RN	University Health Network, University of Toroi	Toronto ON, Canada	Collaborator	
Janice	Williams			University Health Network, University of Toroi	Toronto ON, Canada	Collaborator	
Tim	Stewart			University Health Network, University of Toroi	Toronto ON, Canada	Collaborator	
Karen	Misquitta		RN	University Health Network, University of Toroi	Toronto ON, Canada	Collaborator	
Aditya	Bharatha		MD	St. Michael’s Hospital, University of Toronto	Toronto, ON, Canada	Site PI	
Daniel H.	Selchen		MD, FRCPC	St. Michael’s Hospital, University of Toronto	Toronto, ON, Canada	Collaborator	
Thomas R.	Marotta		MD, FRCPC	St. Michael’s Hospital, University of Toronto	Toronto, ON, Canada	Collaborator	
Dipanka	Sarma		MBBS	St. Michael’s Hospital, University of Toronto	Toronto, ON, Canada	Collaborator	
Walter J.	Montanera		MD, FRCPC	St. Michael’s Hospital, University of Toronto	Toronto, ON, Canada	Collaborator	
Julian	Spears		MD, SM, FRCSC, FACS	St. Michael’s Hospital, University of Toronto	Toronto, ON, Canada	Collaborator	
Gustavo	Saposnik		MD, MSc, FRCPC	St. Michael’s Hospital, University of Toronto	Toronto, ON, Canada	Collaborator	
Pawel	Kostyrko		MD, MSc	St. Michael’s Hospital, University of Toronto	Toronto, ON, Canada	Collaborator	
Yangmei	Li		PhD, CCRC, CCRP	St. Michael’s Hospital, University of Toronto	Toronto, ON, Canada	Collaborator	

*Indicates required information. Only first name, last name, and suffix will appear in PubMed.

*First Name and Middle Initial(s)	*Last Name	*Suffix (eg, Jr, III)	Academic Degrees	Institution	Location (city, state/province, country)	Role or Contribution, eg, chair, principal investigator	Group (if more than 1 Group listed in the byline) and/or Subgroup (eg, Steering Committee)
Carmen	Parra-Farinas		MD	St. Michael’s Hospital, University of Toronto	Toronto, ON, Canada	Collaborator	
Jose Danilo Bengzon	Diestro		MD	St. Michael’s Hospital, University of Toronto	Toronto, ON, Canada	Collaborator	
Marie-Christine	Camden		MD	CHU de Québec - Enfant-Jésus Hospital, Laval U	Québec City, QC, Canada	Site PI	
Steve	Verreault		MD, FRCPC	CHU de Québec - Enfant-Jésus Hospital, Laval U	Québec City, QC, Canada	Collaborator	
Ariane	Mackey		MD, FRCPC	CHU de Québec - Enfant-Jésus Hospital, Laval U	Québec City, QC, Canada	Collaborator	
Marie-Eve	Audet		MD, FRCPC	CHU de Québec - Enfant-Jésus Hospital, Laval U	Québec City, QC, Canada	Collaborator	
Geneviève	Milot		MD, FRCPC	CHU de Québec - Enfant-Jésus Hospital, Laval U	Québec City, QC, Canada	Collaborator	
Pascale	Lavoie		MD, FRCPC	CHU de Québec - Enfant-Jésus Hospital, Laval U	Québec City, QC, Canada	Collaborator	
Jean-Luc	Gariépy		MD, FRCPC	CHU de Québec - Enfant-Jésus Hospital, Laval U	Québec City, QC, Canada	Collaborator	
Karine	Collard			CHU de Québec - Enfant-Jésus Hospital, Laval U	Québec City, QC, Canada	Collaborator	
Annette	Haché			CHU de Québec - Enfant-Jésus Hospital, Laval U	Québec City, QC, Canada	Collaborator	
Demetrios J.	Sahlas		MD	McMaster University	Hamilton, ON, Canada	Site PI	
Ahmed M.	Alshanbari		MBBS	McMaster University	Hamilton, ON, Canada	Collaborator	
Josephine	Baldwin			McMaster University	Hamilton, ON, Canada	Collaborator	
Luciana	Catanese		MD	McMaster University	Hamilton, ON, Canada	Collaborator	
Bing Y.	Chen		MD	McMaster University	Hamilton, ON, Canada	Collaborator	
Danielle de	Sa Boasquevisque		MD	McMaster University	Hamilton, ON, Canada	Collaborator	
Aviraj S.	Deshmukh		MBBS	McMaster University	Hamilton, ON, Canada	Collaborator	
Josrdan M.	Knapman		MSc	McMaster University	Hamilton, ON, Canada	Collaborator	
Shana	Lamers		MSc	McMaster University	Hamilton, ON, Canada	Collaborator	
Ramiro A.	Larrazabal		MD	McMaster University	Hamilton, ON, Canada	Collaborator	
Susan V.	MacMillan		MSc	McMaster University	Hamilton, ON, Canada	Collaborator	
Lauren M.	Mai		MD	McMaster University	Hamilton, ON, Canada	Collaborator	
Alicia M.	Mattai		MD	McMaster University	Hamilton, ON, Canada	Collaborator	
Sumiti	Nayar		MD	McMaster University	Hamilton, ON, Canada	Collaborator	
Kuan H.	Ng		MBBS	McMaster University	Hamilton, ON, Canada	Collaborator	
Wieslaw J.	Oczkowski		MD	McMaster University	Hamilton, ON, Canada	Collaborator	
Kanjana S.	Perera		MBBS	McMaster University	Hamilton, ON, Canada	Collaborator	
Mays A. K.	Shawawrah		MBBS	McMaster University	Hamilton, ON, Canada	Collaborator	
Ashkan	Shoamanesh		MD	McMaster University	Hamilton, ON, Canada	Collaborator	
Gita	Sobhi		BSc	McMaster University	Hamilton, ON, Canada	Collaborator	
Nabeel M.	Syed		MD	McMaster University	Hamilton, ON, Canada	Collaborator	

Supplemental Online Content: Nonauthor Collaborators

*Indicates required information. Only first name, last name, and suffix will appear in PubMed.

***First Name and Middle Initial(s)**	***Last Name**	***Suffix (eg, Jr, III)**	Academic Degrees	Institution	Location (city, state/province, country)	Role or Contribution, eg, chair, principal investigator	Group (if more than 1 Group listed in the byline) and/or Subgroup (eg, Steering Committee)
Noran Y.	Yaher		MBBS	McMaster University	Hamilton, ON, Canada	Collaborator	
Cheyanne	Vandervelde		BSc	McMaster University	Hamilton, ON, Canada	Collaborator	
Di	Wang		Pharm D	McMaster University	Hamilton, ON, Canada	Collaborator	
Thalia S.	Field		MD, FRCPC, MHSc	University of British Columbia	Vancouver, BC, Canada	Site PI	
Stephen C.	Van Gaal		MD, FRCPC	University of British Columbia	Vancouver, BC, Canada	Collaborator	
Philip A.	Teal		MD, FRCPC	University of British Columbia	Vancouver, BC, Canada	Collaborator	
Oscar R.	Benavente		MD, FRCPC	University of British Columbia	Vancouver, BC, Canada	Collaborator	
Laura K.	Wilson		MD, FRCPC	University of British Columbia	Vancouver, BC, Canada	Collaborator	
Sharanpal K.	Mann		MD, FRCPC	University of British Columbia	Vancouver, BC, Canada	Collaborator	
Samuel	Yip		PhD, MD, FRCPC	University of British Columbia	Vancouver, BC, Canada	Collaborator	
Colleen B.	Murphy		MD, FRCPC	University of British Columbia	Vancouver, BC, Canada	Collaborator	
Manraj K.S.	Heran		MD, FRCPC	University of British Columbia	Vancouver, BC, Canada	Collaborator	
Axel	Rohr		MD, PhD	University of British Columbia	Vancouver, BC, Canada	Collaborator	
Fabio	Settecase		MD, MSc, FRCPC	University of British Columbia	Vancouver, BC, Canada	Collaborator	
Gary J.	Redekop		MD, MSc, FRCSC	University of British Columbia	Vancouver, BC, Canada	Collaborator	
Charles S.	Haw		MD, MSc, FRCSC	University of British Columbia	Vancouver, BC, Canada	Collaborator	
Genoveva	Maclean		RN	University of British Columbia	Vancouver, BC, Canada	Collaborator	
Karina	Murray		RN	University of British Columbia	Vancouver, BC, Canada	Collaborator	
Rosalin	Chiu		RN	University of British Columbia	Vancouver, BC, Canada	Collaborator	
Alana M.	Flexman		MD, FRCPC	University of British Columbia	Vancouver, BC, Canada	Collaborator	
Eilidh	Strecha		RN	University of British Columbia	Vancouver, BC, Canada	Collaborator	
Yolanda	Gayton		RN	University of British Columbia	Vancouver, BC, Canada	Collaborator	
Judy	Yip		BSc (Pharm), ACPR	University of British Columbia	Vancouver, BC, Canada	Collaborator	
Richard H.	Swartz		MD, PhD	Sunnybrook Health Sciences Centre, University	Toronto ON, Canada	Site PI	
Mark I.	Boulos		MD	Sunnybrook Health Sciences Centre, University	Toronto ON, Canada	Collaborator	
David J.	Gladstone		MD, PhD	Sunnybrook Health Sciences Centre, University	Toronto ON, Canada	Collaborator	
Amy Y.	Yu		MD	Sunnybrook Health Sciences Centre, University	Toronto ON, Canada	Collaborator	

Supplemental Online Content: Nonauthor Collaborators

*Indicates required information. Only first name, last name, and suffix will appear in PubMed.

*First Name and Middle Initial(s)	*Last Name	*Suffix (eg, Jr, III)	Academic Degrees	Institution	Location (city, state/province, country)	Role or Contribution, eg, chair, principal investigator	Group (if more than 1 Group listed in the byline) and/or Subgroup (eg, Steering Committee)
Julia J.	Hopyan		MBBS	Sunnybrook Health Sciences Centre, University	Toronto ON, Canada	Collaborator	
Houman	Khosravani		MD, PhD	Sunnybrook Health Sciences Centre, University	Toronto ON, Canada	Collaborator	
Herbert A.	Manosalva Alzate		MD	Sunnybrook Health Sciences Centre, University	Toronto ON, Canada	Collaborator	
Alisia	Southwell		HBSc	Sunnybrook Health Sciences Centre, University	Toronto ON, Canada	Collaborator	
Maneesha	Kamra		MD	Sunnybrook Health Sciences Centre, University	Toronto ON, Canada	Collaborator	
Kaitlyn N.	Lopes		BMSc	Sunnybrook Health Sciences Centre, University	Toronto ON, Canada	Collaborator	
Arunima	Kapoor		MSc	Sunnybrook Health Sciences Centre, University	Toronto ON, Canada	Collaborator	
Leodante	da Costa		MD	Sunnybrook Health Sciences Centre, University	Toronto ON, Canada	Collaborator	
Victor X. D.	Yang		MD, PhD	Sunnybrook Health Sciences Centre, University	Toronto ON, Canada	Collaborator	
Erin E.	Dyer		MD	Sunnybrook Health Sciences Centre, University	Toronto ON, Canada	Collaborator	
Ruchir A.	Shah		MD	Erlanger Hospital	Chattanooga, TN, US	Site PI	
Brenda M.	Knowles		MSN, RN	Erlanger Hospital	Chattanooga, TN, US	Collaborator	
Jennifer	Nichols		MSN, AGACNP-BC	Erlanger Hospital	Chattanooga, TN, US	Collaborator	
Jennifer L.	Patterson		RN, MSN, ACNP-BC, CCRN, FHM	Erlanger Hospital	Chattanooga, TN, US	Collaborator	
Krista A.	Cope		Pharm D, RD, LDN	Erlanger Hospital	Chattanooga, TN, US	Collaborator	
Lee	Dickerson		aPT	Erlanger Hospital	Chattanooga, TN, US	Collaborator	
Katrina K.	Barton		CCRC	Erlanger Hospital	Chattanooga, TN, US	Collaborator	
Kimberly D.	Gray		CRC	Erlanger Hospital	Chattanooga, TN, US	Collaborator	
Ticey N.	Massengale		CRC	Erlanger Hospital	Chattanooga, TN, US	Collaborator	
Melanie G.	Miller		RN, CRNI	Erlanger Hospital	Chattanooga, TN, US	Collaborator	
Cindy	Simpson		BSN, RN	Erlanger Hospital	Chattanooga, TN, US	Collaborator	
Sabrina	Walker		MSN, RN-BC	Erlanger Hospital	Chattanooga, TN, US	Collaborator	
Chlin R.	Tennyson-Yemm		BSN RN	Erlanger Hospital	Chattanooga, TN, US	Collaborator	
Donald F.	Frei		MD	Swedish Medical Center- Colorado Neurologic	Denver, CO, US	Site PI	
Richard J,	Bellon		MD	Swedish Medical Center- Colorado Neurologic	Denver, CO, US	Collaborator	
Benjamin N.	Atchie		DO	Swedish Medical Center- Colorado Neurologic	Denver, CO, US	Collaborator	
Ian A.	Kaminsky		MD	Swedish Medical Center- Colorado Neurologic	Denver, CO, US	Collaborator	

Supplemental Online Content: Nonauthor Collaborators

*Indicates required information. Only first name, last name, and suffix will appear in PubMed.

*First Name and Middle Initial(s)	*Last Name	*Suffix (eg, Jr, III)	Academic Degrees	Institution	Location (city, state/province, country)	Role or Contribution, eg, chair, principal investigator	Group (if more than 1 Group listed in the byline) and/or Subgroup (eg, Steering Committee)
Duandelyn C.	Wilson		NP	Swedish Medical Center- Colorado Neurologic	Denver, CO, US	Collaborator	
Nicolle L.	Schraeder		NP	Swedish Medical Center- Colorado Neurologic	Denver, CO, US	Collaborator	
Eric J.	Arias		MD	Swedish Medical Center- Colorado Neurologic	Denver, CO, US	Collaborator	
Lisa M.	Kodis		MSN, RN	Swedish Medical Center- Colorado Neurologic	Denver, CO, US	Collaborator	
Mark E.	Talley		MS	Swedish Medical Center- Colorado Neurologic	Denver, CO, US	Collaborator	
Alex A.	Edinger		BS	Swedish Medical Center- Colorado Neurologic	Denver, CO, US	Collaborator	
Tiffany C.	Talley		MS	Swedish Medical Center- Colorado Neurologic	Denver, CO, US	Collaborator	
Ita P.	Dempsey		RN	Swedish Medical Center- Colorado Neurologic	Denver, CO, US	Collaborator	
Laurie A.	Williams		RN	Swedish Medical Center- Colorado Neurologic	Denver, CO, US	Collaborator	
Scott A.	Williams		Pharm D, BCOP	Swedish Medical Center- Colorado Neurologic	Denver, CO, US	Collaborator	
Sonny	Kupniewski		Pharm D, BCPS	Swedish Medical Center- Colorado Neurologic	Denver, CO, US	Collaborator	
Brad K.	Fasbinder		BS	Swedish Medical Center- Colorado Neurologic	Denver, CO, US	Collaborator	
Joanna M.	Snead		MS, CCRP	Swedish Medical Center- Colorado Neurologic	Denver, CO, US	Collaborator	
Hana	Choe		MD	Neurosciences Institute, Abington Jefferson H	Philadelphia, PA, US	Site PI	
Larami	Mackenzie		MD	Neurosciences Institute, Abington Jefferson H	Philadelphia, PA, US	Collaborator	
David C.	Weisman		MD	Neurosciences Institute, Abington Jefferson H	Philadelphia, PA, US	Collaborator	
Osman S.	Kozak		MD	Neurosciences Institute, Abington Jefferson H	Philadelphia, PA, US	Collaborator	
Qaisar A.	Shah		MD	Neurosciences Institute, Abington Jefferson H	Philadelphia, PA, US	Collaborator	
Dan	Gzesh		MD	Neurosciences Institute, Abington Jefferson H	Philadelphia, PA, US	Collaborator	
Kandan	Kulandaivel		MD	Neurosciences Institute, Abington Jefferson H	Philadelphia, PA, US	Collaborator	
John S.	Khoury		MD	Neurosciences Institute, Abington Jefferson H	Philadelphia, PA, US	Collaborator	
Brad	Klein		MD, MBA	Neurosciences Institute, Abington Jefferson H	Philadelphia, PA, US	Collaborator	
Patricia A.	Bussinger		MBA, MSN,AGACNP-BC, CRNP	Neurosciences Institute, Abington Jefferson H	Philadelphia, PA, US	Collaborator	
Lisa R.	Griffin		AGACNP-BC, CRNP	Neurosciences Institute, Abington Jefferson H	Philadelphia, PA, US	Collaborator	
Ashley L.	DePalmo		BA, CRC	Neurosciences Institute, Abington Jefferson H	Philadelphia, PA, US	Collaborator	
Cynthia	Oliva		PharmD	Neurosciences Institute, Abington Jefferson H	Philadelphia, PA, US	Collaborator	
Ashutosh	Jadhav		MD	UPMC Medical Centre	Pittsburgh, PA, US	Site PI	
Tudor	Jovin		MD	UPMC Medical Centre	Pittsburgh, PA, US	Collaborator	

Supplemental Online Content: Nonauthor Collaborators

*Indicates required information. Only first name, last name, and suffix will appear in PubMed.

*First Name and Middle Initial(s)	*Last Name	*Suffix (eg, Jr, III)	Academic Degrees	Institution	Location (city, state/province, country)	Role or Contribution, eg, chair, principal investigator	Group (if more than 1 Group listed in the byline) and/or Subgroup (eg, Steering Committee)
Cynthia	Kenmuir		MD	UPMC Medical Centre	Pittsburgh, PA, US	Collaborator	
Brian	Jankowitz		MD	UPMC Medical Centre	Pittsburgh, PA, US	Collaborator	
Bradley	Gross		MD	UPMC Medical Centre	Pittsburgh, PA, US	Collaborator	
Marcelo	Rocha		MD	UPMC Medical Centre	Pittsburgh, PA, US	Collaborator	
Matthew	Starr		MD	UPMC Medical Centre	Pittsburgh, PA, US	Collaborator	
Merritt	Brown		MD	UPMC Medical Centre	Pittsburgh, PA, US	Collaborator	
Chrstine	Hawkes		MD	UPMC Medical Centre	Pittsburgh, PA, US	Collaborator	
Kavit	Shah		MD	UPMC Medical Centre	Pittsburgh, PA, US	Collaborator	
Danoushka	Tememe		MD	UPMC Medical Centre	Pittsburgh, PA, US	Collaborator	
Gregory	Walker		MD	UPMC Medical Centre	Pittsburgh, PA, US	Collaborator	
Pratit	Patel		MD	UPMC Medical Centre	Pittsburgh, PA, US	Collaborator	
Bradley	Klein		MD	UPMC Medical Centre	Pittsburgh, PA, US	Collaborator	
Habibullah	Ziayee		MD	UPMC Medical Centre	Pittsburgh, PA, US	Collaborator	
Kaustubh	Limaye		MD	UPMC Medical Centre	Pittsburgh, PA, US	Collaborator	
Lisa	Baxendell		BSN	UPMC Medical Centre	Pittsburgh, PA, US	Collaborator	
Vicki	Glichrist		BSN	UPMC Medical Centre	Pittsburgh, PA, US	Collaborator	
Patricia	Feineigle		BSN,PhD	UPMC Medical Centre	Pittsburgh, PA, US	Collaborator	
Kelsea	Toseki Haibach		BSN	UPMC Medical Centre	Pittsburgh, PA, US	Collaborator	
Cathy	Van Every		BSN	UPMC Medical Centre	Pittsburgh, PA, US	Collaborator	
Shasvat	Desai			UPMC Medical Centre	Pittsburgh, PA, US	Collaborator	
Maryam	Zulfiqar			UPMC Medical Centre	Pittsburgh, PA, US	Collaborator	
Linda	Gibson			UPMC Medical Centre	Pittsburgh, PA, US	Collaborator	
Sean	Barrett			UPMC Medical Centre	Pittsburgh, PA, US	Collaborator	
David	Turkel-Parrella		MD	NYU School of Medicine	Brooklyn, NY, US	Site PI	
Karthikeyan	Arcot		MD	NYU School of Medicine	Brooklyn, NY, US	Collaborator	
Steven	DiCrescento		RPh	NYU School of Medicine	Brooklyn, NY, US	Collaborator	
Jeffrey	Farkas		MD	NYU School of Medicine	Brooklyn, NY, US	Collaborator	
Gregory	Filipowski		RPh	NYU School of Medicine	Brooklyn, NY, US	Collaborator	
Jennifer	Frontera		MD	NYU School of Medicine	Brooklyn, NY, US	Collaborator	
Danielle	Joset		PharmD	NYU School of Medicine	Brooklyn, NY, US	Collaborator	
Jeremy	Liff		MD	NYU School of Medicine	Brooklyn, NY, US	Collaborator	
Erica	Scher		RN, MPH	NYU School of Medicine	Brooklyn, NY, US	Collaborator	
Cameron G.	McDougall		MD	Swedish Neurological Institute	Seattle, WA, US	Site PI	
Cheryl E.	Kelly		BSN, CNRN	Swedish Neurological Institute	Seattle, WA, US	Collaborator	

Supplemental Online Content: Nonauthor Collaborators

*Indicates required information. Only first name, last name, and suffix will appear in PubMed.

*First Name and Middle Initial(s)	*Last Name	*Suffix (eg, Jr, III)	Academic Degrees	Institution	Location (city, state/province, country)	Role or Contribution, eg, chair, principal investigator	Group (if more than 1 Group listed in the byline) and/or Subgroup (eg, Steering Committee)
Akshal S.	Patel		MD	Swedish Neurological Institute	Seattle, WA, US	Collaborator	
Stephen J.	Monteith		MD	Swedish Neurological Institute	Seattle, WA, US	Collaborator	
Sidney	Starkman		MD	UCLA Comprehensive Stroke Center, Universit	Los Angeles, Los Angeles,	Site PI	
Kunakorn C.	Atchaneeyasakul		MD	UCLA Comprehensive Stroke Center, Universit	Los Angeles, Los Angeles,	Collaborator	
Adrian M.	Burgos		MD	UCLA Comprehensive Stroke Center, Universit	Los Angeles, Los Angeles,	Collaborator	
Janice	Anne Y. Chua		BSN, CNRN	UCLA Comprehensive Stroke Center, Universit	Los Angeles, Los Angeles,	Collaborator	
Nathan D.	Gaines		MD	UCLA Comprehensive Stroke Center, Universit	Los Angeles, Los Angeles,	Collaborator	
Ileana D.	Grunberg		RN	UCLA Comprehensive Stroke Center, Universit	Los Angeles, Los Angeles,	Collaborator	
Judy	Guzy		RN	UCLA Comprehensive Stroke Center, Universit	Los Angeles, Los Angeles,	Collaborator	
Zuolu	Liu		MD	UCLA Comprehensive Stroke Center, Universit	Los Angeles, Los Angeles,	Collaborator	
Neil D.	Maluste		MD	UCLA Comprehensive Stroke Center, Universit	Los Angeles, Los Angeles,	Collaborator	
Lucas	Ramirez		MD	UCLA Comprehensive Stroke Center, Universit	Los Angeles, Los Angeles,	Collaborator	
Latisha K.	Sharma		MD	UCLA Comprehensive Stroke Center, Universit	Los Angeles, Los Angeles,	Collaborator	
Donald	Heck		MD	Forsyth Medical Center	Winston-Salem, NC, US	Site PI	
Morry	Brown		MD, PhD	Forsyth Medical Center	Winston-Salem, NC, US	Collaborator	
Colin	McDonald		MD	Forsyth Medical Center	Winston-Salem, NC, US	Collaborator	
Mateo	Calderon		MD	Forsyth Medical Center	Winston-Salem, NC, US	Collaborator	
Mitch	Hargis		MD	Forsyth Medical Center	Winston-Salem, NC, US	Collaborator	
Christina	Roels		Pharm D	Forsyth Medical Center	Winston-Salem, NC, US	Collaborator	
Prabhu	Emmady		MD	Forsyth Medical Center	Winston-Salem, NC, US	Collaborator	
Talat	Alvi		MD	Forsyth Medical Center	Winston-Salem, NC, US	Collaborator	
Mahesh V.	Jayaraman		MD	Warren Alpert School of Medicine at Brown U	Providence, RI, US	Collaborator	
Shawna M.	Cutting		MD	Warren Alpert School of Medicine at Brown U	Providence, RI, US	Collaborator	
Wendy J.	Smith		BS, RTRCV	Warren Alpert School of Medicine at Brown U	Providence, RI, US	Collaborator	
Susan M.	Foley		RTRCV	Warren Alpert School of Medicine at Brown U	Providence, RI, US	Collaborator	
Gino A.	Paolucci		NP	Warren Alpert School of Medicine at Brown U	Providence, RI, US	Collaborator	
Richard A.	Haas		MD	Warren Alpert School of Medicine at Brown U	Providence, RI, US	Collaborator	
Katie A.	Quinn		PA	Warren Alpert School of Medicine at Brown U	Providence, RI, US	Collaborator	
Lindsey R.	Fuller		PA	Warren Alpert School of Medicine at Brown U	Providence, RI, US	Collaborator	
Rebecca E.	Brierley		PA	Warren Alpert School of Medicine at Brown U	Providence, RI, US	Collaborator	
Christina C.	Watkins		NP	Warren Alpert School of Medicine at Brown U	Providence, RI, US	Collaborator	
Nicole C.	Demir		NP	Warren Alpert School of Medicine at Brown U	Providence, RI, US	Collaborator	

Supplemental Online Content: Nonauthor Collaborators

*Indicates required information. Only first name, last name, and suffix will appear in PubMed.

*First Name and Middle Initial(s)	*Last Name	*Suffix (eg, Jr, III)	Academic Degrees	Institution	Location (city, state/province, country)	Role or Contribution, eg, chair, principal investigator	Group (if more than 1 Group listed in the byline) and/or Subgroup (eg, Steering Committee)
George A.	Lopez		MD, PhD	Swedish Neurological Institute	Seattle, WA, US	Site PI	
Alejandro	Vargas		MD	Rush University Medical Center	Chicago, IL, US	Collaborator	
Nicholas	Osteraas		MD	Rush University Medical Center	Chicago, IL, US	Collaborator	
Becky	Holtz		RN	Rush University Medical Center	Chicago, IL, US	Collaborator	
Eric	Sauvageau		MD	Lyerly Neurosurgery, Baptist Hospital	Jacksonville, FL, US	Site PI	
Amin	Aghaebrahim		MD	Lyerly Neurosurgery, Baptist Hospital	Jacksonville, FL, US	Collaborator	
Mohamad	Chmayssani		MD	Lyerly Neurosurgery, Baptist Hospital	Jacksonville, FL, US	Collaborator	
Neeraj S.	Naval		MD	Lyerly Neurosurgery, Baptist Hospital	Jacksonville, FL, US	Collaborator	
Jason S.	Day		MD	Lyerly Neurosurgery, Baptist Hospital	Jacksonville, FL, US	Collaborator	
Scott M.	Dellorso		MD	Lyerly Neurosurgery, Baptist Hospital	Jacksonville, FL, US	Collaborator	
Benjamin J.	Ludwig		MD	Lyerly Neurosurgery, Baptist Hospital	Jacksonville, FL, US	Collaborator	
Derek J.	Schemmel		MD	Lyerly Neurosurgery, Baptist Hospital	Jacksonville, FL, US	Collaborator	
Nancy	Ebreo		CCRC, RRT	Lyerly Neurosurgery, Baptist Hospital	Jacksonville, FL, US	Collaborator	
Karen	Bell		BSH	Lyerly Neurosurgery, Baptist Hospital	Jacksonville, FL, US	Collaborator	
Lanai	Lewis		CRC	Lyerly Neurosurgery, Baptist Hospital	Jacksonville, FL, US	Collaborator	
Marjorie	Delucia		PharmD	Lyerly Neurosurgery, Baptist Hospital	Jacksonville, FL, US	Collaborator	
Diogo	Haussen		MD	Emory University School of Medicine, Grady M	Atlanta, GA, US	Collaborator	
Michael	Frankel		MD	Emory University School of Medicine, Grady M	Atlanta, GA, US	Collaborator	
Nirav	Bhatt		MD	Emory University School of Medicine, Grady M	Atlanta, GA, US	Collaborator	
Nicolas	Bianchi		MD	Emory University School of Medicine, Grady M	Atlanta, GA, US	Collaborator	
Aaron	Anderson		MD	Emory University School of Medicine, Grady M	Atlanta, GA, US	Collaborator	
Samir	Belagaje		MD	Emory University School of Medicine, Grady M	Atlanta, GA, US	Collaborator	
Bernardo	Liberato		MD	Emory University School of Medicine, Grady M	Atlanta, GA, US	Collaborator	
Srikant	Rangaraju		MD	Emory University School of Medicine, Grady M	Atlanta, GA, US	Collaborator	
Alhamza R.	Al-Bayati		MD	Emory University School of Medicine, Grady M	Atlanta, GA, US	Collaborator	
Jonathan	Grossber		MD	Emory University School of Medicine, Grady M	Atlanta, GA, US	Collaborator	
Leah P.	Craft		RN, BSN, CCRC	Emory University School of Medicine, Grady M	Atlanta, GA, US	Collaborator	
Kiva	Schindler		RN, CCRC	Emory University School of Medicine, Grady M	Atlanta, GA, US	Collaborator	
Erin	Schaad		CRC	Emory University School of Medicine, Grady M	Atlanta, GA, US	Collaborator	
Meagan	Schultz		CRC	Emory University School of Medicine, Grady M	Atlanta, GA, US	Collaborator	

Supplemental Online Content: Nonauthor Collaborators

*Indicates required information. Only first name, last name, and suffix will appear in PubMed.

*First Name and Middle Initial(s)	*Last Name	*Suffix (eg, Jr, III)	Academic Degrees	Institution	Location (city, state/province, country)	Role or Contribution, eg, chair, principal investigator	Group (if more than 1 Group listed in the byline) and/or Subgroup (eg, Steering Committee)
Lorretta J.	Sutherly		MS, BSN, RN-BC, SCRN, NVRN, CCRC	Emory University School of Medicine, Grady M	Atlanta, GA, US	Collaborator	
Shannon	Doppelheuer		CCRC	Emory University School of Medicine, Grady M	Atlanta, GA, US	Collaborator	
Jacquelyn	Charlton		CRC	Emory University School of Medicine, Grady M	Atlanta, GA, US	Collaborator	
Jason	Faggard		RN	Emory University School of Medicine, Grady M	Atlanta, GA, US	Collaborator	
Zuzana	Barbret		RN	Emory University School of Medicine, Grady M	Atlanta, GA, US	Collaborator	
Ethan	DukSoo Han		RN	Emory University School of Medicine, Grady M	Atlanta, GA, US	Collaborator	
Robin	Walters		RN	Emory University School of Medicine, Grady M	Atlanta, GA, US	Collaborator	
Jaydevsinh	Dolia		MD	Emory University School of Medicine, Grady M	Atlanta, GA, US	Collaborator	
Sleiman	El-Jamal		MD	Emory University School of Medicine, Grady M	Atlanta, GA, US	Collaborator	
Brendan	Eby		MD	Emory University School of Medicine, Grady M	Atlanta, GA, US	Collaborator	
Yasir	Saleem		MD	Emory University School of Medicine, Grady M	Atlanta, GA, US	Collaborator	
Harrison	Pearl		MD	Emory University School of Medicine, Grady M	Atlanta, GA, US	Collaborator	
Kishan	Patel		MD	Emory University School of Medicine, Grady M	Atlanta, GA, US	Collaborator	
Haseeb	Rahman		MD	Emory University School of Medicine, Grady M	Atlanta, GA, US	Collaborator	
Reema	Butt		MD	Emory University School of Medicine, Grady M	Atlanta, GA, US	Collaborator	
Stephen	English		MD	Emory University School of Medicine, Grady M	Atlanta, GA, US	Collaborator	
Ajit S.	Puri		MD	University of Massachusetts Medical Center	Worcester, MA, US	Site PI	
Mary	Howk		MS	University of Massachusetts Medical Center	Worcester, MA, US	Collaborator	
Jasmeet	Singh		MD	University of Massachusetts Medical Center	Worcester, MA, US	Collaborator	
Francesco	Massari		MD, PhD	University of Massachusetts Medical Center	Worcester, MA, US	Collaborator	
Katyucia	DeMacedo Rodrigues		MD	University of Massachusetts Medical Center	Worcester, MA, US	Collaborator	
Anna L.	Kuhn		MD, PhD	University of Massachusetts Medical Center	Worcester, MA, US	Collaborator	
Joey D.	English		MD	California Pacific Medical Center, Sutter Healt	San Francisco, CA, US	Site PI	
Nobl	Barazangi		MD	California Pacific Medical Center, Sutter Healt	San Francisco, CA, US	Collaborator	
Nick	Telischak		MD	California Pacific Medical Center, Sutter Healt	San Francisco, CA, US	Collaborator	
Warren	Kim		MD	California Pacific Medical Center, Sutter Healt	San Francisco, CA, US	Collaborator	
Josh	Ross		RN	California Pacific Medical Center, Sutter Healt	San Francisco, CA, US	Collaborator	
Nata	DeVole		RN	California Pacific Medical Center, Sutter Healt	San Francisco, CA, US	Collaborator	
Jessica	Redford		RN	California Pacific Medical Center, Sutter Healt	San Francisco, CA, US	Collaborator	

Supplemental Online Content: Nonauthor Collaborators

*Indicates required information. Only first name, last name, and suffix will appear in PubMed.

*First Name and Middle Initial(s)	*Last Name	*Suffix (eg, Jr, III)	Academic Degrees	Institution	Location (city, state/province, country)	Role or Contribution, eg, chair, principal investigator	Group (if more than 1 Group listed in the byline) and/or Subgroup (eg, Steering Committee)
Melina	Ferreira			California Pacific Medical Center, Sutter Healtl	San Francisco, CA, US	Collaborator	
Katie	Ponting			California Pacific Medical Center, Sutter Healtl	San Francisco, CA, US	Collaborator	
Helen	Shen			California Pacific Medical Center, Sutter Healtl	San Francisco, CA, US	Collaborator	
Ann	Bedenk		RN	California Pacific Medical Center, Sutter Healtl	San Francisco, CA, US	Collaborator	
Susila	Patel		RN	California Pacific Medical Center, Sutter Healtl	San Francisco, CA, US	Collaborator	
Julia	Fernandess		RN	California Pacific Medical Center, Sutter Healtl	San Francisco, CA, US	Collaborator	
Michael	Ke		MD	California Pacific Medical Center, Sutter Healtl	San Francisco, CA, US	Collaborator	
Illanit	Spokoyny		MD	California Pacific Medical Center, Sutter Healtl	San Francisco, CA, US	Collaborator	
Billy	Gao		MD	California Pacific Medical Center, Sutter Healtl	San Francisco, CA, US	Collaborator	
David	Tong		MD	California Pacific Medical Center, Sutter Healtl	San Francisco, CA, US	Collaborator	
Charlene	Chen		MD	California Pacific Medical Center, Sutter Healtl	San Francisco, CA, US	Collaborator	
Christine	Wong		MD	California Pacific Medical Center, Sutter Healtl	San Francisco, CA, US	Collaborator	
Jessica	Choe		MD	California Pacific Medical Center, Sutter Healtl	San Francisco, CA, US	Collaborator	
Coleman O	Martin		MD	Saint Luke’s Hospital of Kansas City	Kansas City, MO, US	Site PI	
Debbie V.	Summers		MSN	Saint Luke’s Hospital of Kansas City	Kansas City, MO, US	Collaborator	
Christine M.	Boutwell		MD	Saint Luke’s Hospital of Kansas City	Kansas City, MO, US	Collaborator	
Karin E.	Olds		MD	Saint Luke’s Hospital of Kansas City	Kansas City, MO, US	Collaborator	
Suzanne C.	Crandall		MD	Saint Luke’s Hospital of Kansas City	Kansas City, MO, US	Collaborator	
John D.	Eatman		MD	Saint Luke’s Hospital of Kansas City	Kansas City, MO, US	Collaborator	
Naveed	Akhtar		MD	Saint Luke’s Hospital of Kansas City	Kansas City, MO, US	Collaborator	
William	Holloway		MD	Saint Luke’s Hospital of Kansas City	Kansas City, MO, US	Collaborator	
Jared S.	Halpin		MD	Saint Luke’s Hospital of Kansas City	Kansas City, MO, US	Collaborator	
Brett	Donegan		MD	Saint Luke’s Hospital of Kansas City	Kansas City, MO, US	Collaborator	
Joseph L.	Schindler		MD	Yale University School of Medicine	New Haven, CT, US	Site PI	
Daiv Y.	Hwang		MD	Yale University School of Medicine	New Haven, CT, US	Collaborator	
Emily J.	Gilmore		MD	Yale University School of Medicine	New Haven, CT, US	Collaborator	
Nils	Petersen		MD	Yale University School of Medicine	New Haven, CT, US	Collaborator	
Kevin N.	Sheth		MD	Yale University School of Medicine	New Haven, CT, US	Collaborator	
Stacy Y.	Brown		MD	Yale University School of Medicine	New Haven, CT, US	Collaborator	
Rachel B.	Beekman		MD	Yale University School of Medicine	New Haven, CT, US	Collaborator	
Benjamin P.	George		MD	Yale University School of Medicine	New Haven, CT, US	Collaborator	
Firas	Kaddouh		MD	Yale University School of Medicine	New Haven, CT, US	Collaborator	
Guido J.	Falcone		MD	Yale University School of Medicine	New Haven, CT, US	Collaborator	
Charles R.	Wira		MD	Yale University School of Medicine	New Haven, CT, US	Collaborator	

Supplemental Online Content: Nonauthor Collaborators

*Indicates required information. Only first name, last name, and suffix will appear in PubMed.

*First Name and Middle Initial(s)	*Last Name	*Suffix (eg, Jr, III)	Academic Degrees	Institution	Location (city, state/province, country)	Role or Contribution, eg, chair, principal investigator	Group (if more than 1 Group listed in the byline) and/or Subgroup (eg, Steering Committee)
Charles C.	Matouk		MD	Yale University School of Medicine	New Haven, CT, US	Collaborator	
Caitlin	Loomis		MD	Yale University School of Medicine	New Haven, CT, US	Collaborator	
Hardik	Amin		MD	Yale University School of Medicine	New Haven, CT, US	Collaborator	
Hans Christoph	Stretz		MD	Yale University School of Medicine	New Haven, CT, US	Collaborator	
Reshma	Narula		MD	Yale University School of Medicine	New Haven, CT, US	Collaborator	
Adam S.	Jasne		MD	Yale University School of Medicine	New Haven, CT, US	Collaborator	
Lauren H.	Sansing		MD	Yale University School of Medicine	New Haven, CT, US	Collaborator	
Tijil	Agarwal		MD	Yale University School of Medicine	New Haven, CT, US	Collaborator	
Sara D.	Jasak		BSN	Yale University School of Medicine	New Haven, CT, US	Collaborator	
Briana D.	Fontaine		BA	Yale University School of Medicine	New Haven, CT, US	Collaborator	
Zachary	King		BA	Yale University School of Medicine	New Haven, CT, US	Collaborator	
Lindsey R.	Kouhn		BA	Yale University School of Medicine	New Haven, CT, US	Collaborator	
Hailey	Orgass		BA	Yale University School of Medicine	New Haven, CT, US	Collaborator	
Megan A.	Leary		BS	Yale University School of Medicine	New Haven, CT, US	Collaborator	
Joan L.	Nye		BS	Yale University School of Medicine	New Haven, CT, US	Collaborator	
Kelsey	Halbert		RN, BSN, MSN, CNL, SCRN, CNRN	Yale University School of Medicine	New Haven, CT, US	Collaborator	
Karin V.	Nystrom		MSN, APRN, FAHA	Yale University School of Medicine	New Haven, CT, US	Collaborator	
Kaile B.	Neuschatz		BSN, SCRN	Yale University School of Medicine	New Haven, CT, US	Collaborator	
Dawn M.	Petrucci		BSN, SCRN	Yale University School of Medicine	New Haven, CT, US	Collaborator	
Anna M.	Coppola		BS, RN	Yale University School of Medicine	New Haven, CT, US	Collaborator	
Treasure A.	Joyce		LVN	Providence Little Company of Mary Medical C	Torrance, CA, US	Collaborator	
Sam Y.	Hou		MD, PhD	Providence Little Company of Mary Medical C	Torrance, CA, US	Collaborator	
Mark S.	Umekubo		Pharm D, BCCP	Providence Little Company of Mary Medical C	Torrance, CA, US	Collaborator	
Catrice M.	Nakamura		MSN, SCRN	Providence Little Company of Mary Medical C	Torrance, CA, US	Collaborator	
Renee M.	Ovando		NP, SCRN	Providence Little Company of Mary Medical C	Torrance, CA, US	Collaborator	
Diana	Zuniga		LVN	Providence Little Company of Mary Medical C	Torrance, CA, US	Collaborator	
Yih Lin	Nien		MD	Providence Little Company of Mary Medical C	Torrance, CA, US	Collaborator	
Fernando Mayor	Basto		MD	Providence Little Company of Mary Medical C	Torrance, CA, US	Collaborator	
Allison E.	Arch		MD	Providence Little Company of Mary Medical C	Torrance, CA, US	Collaborator	

Supplemental Online Content: Nonauthor Collaborators

*Indicates required information. Only first name, last name, and suffix will appear in PubMed.

*First Name and Middle Initial(s)	*Last Name	*Suffix (eg, Jr, III)	Academic Degrees	Institution	Location (city, state/province, country)	Role or Contribution, eg, chair, principal investigator	Group (if more than 1 Group listed in the byline) and/or Subgroup (eg, Steering Committee)
Laura T.	Jong		MD	Providence Little Company of Mary Medical C	Torrance, CA, US	Collaborator	
William A.	Conrad		MD	Providence Little Company of Mary Medical C	Torrance, CA, US	Collaborator	
Tara C.	Abbott		BSN	Providence Little Company of Mary Medical C	Torrance, CA, US	Collaborator	
Jay Z.	Yao		MD	Providence Little Company of Mary Medical C	Torrance, CA, US	Collaborator	
Scott D.	Caganap		MD	Providence Little Company of Mary Medical C	Torrance, CA, US	Collaborator	
John E.	Jordan		MD	Providence Little Company of Mary Medical C	Torrance, CA, US	Collaborator	
George P.	Teitelbaum		MD	Providence Little Company of Mary Medical C	Torrance, CA, US	Collaborator	
Robert J.	Darflinger		MD	Providence Little Company of Mary Medical C	Torrance, CA, US	Collaborator	
Daniel F.	Kelly		MD	Providence Little Company of Mary Medical C	Torrance, CA, US	Collaborator	
Rishi	Gupta		MD	Wellstar Health System, Kennestone Hospital	Marietta, GA, US	Site PI	
Ronald F.	Budzik		MD	Ohio Health, Riverside Methodist Hospital	Columbus, OH, US	Site PI	
Jennifer H.	Czerniak		RN	Ohio Health, Riverside Methodist Hospital	Columbus, OH, US	Collaborator	
Katherine M.	Groezinger		BSN, RN, SCRN	Ohio Health, Riverside Methodist Hospital	Columbus, OH, US	Collaborator	
William J.	Hicks	II	MD	Ohio Health, Riverside Methodist Hospital	Columbus, OH, US	Collaborator	
Omran B.	Kaskar		DO	Ohio Health, Riverside Methodist Hospital	Columbus, OH, US	Collaborator	
Brian S.	Katz		MD	Ohio Health, Riverside Methodist Hospital	Columbus, OH, US	Collaborator	
Aaron I.	Loochtan		DO	Ohio Health, Riverside Methodist Hospital	Columbus, OH, US	Collaborator	
Peter J.	Pema		MD	Ohio Health, Riverside Methodist Hospital	Columbus, OH, US	Collaborator	
Vivek	Rai		MD	Ohio Health, Riverside Methodist Hospital	Columbus, OH, US	Collaborator	
Nirav A.	Vora		MD	Ohio Health, Riverside Methodist Hospital	Columbus, OH, US	Collaborator	
Amanda	Brown		MPH	Ohio Health, Riverside Methodist Hospital	Columbus, OH, US	Collaborator	

*Indicates required information. Only first name, last name, and suffix will appear in PubMed.

*First Name and Middle Initial(s)	*Last Name	*Suffix (eg, Jr, III)	Academic Degrees	Institution	Location (city, state/province, country)	Role or Contribution, eg, chair, principal investigator	Group (if more than 1 Group listed in the byline) and/or Subgroup (eg, Steering Committee)
Diane	Goodman			Ohio Health, Riverside Methodist Hospital	Columbus, OH, US	Collaborator	
Barb	Danenbergs		RPh, CCRP	Ohio Health, Riverside Methodist Hospital	Columbus, OH, US	Collaborator	
Monica	Gossett		Pharm D	Ohio Health, Riverside Methodist Hospital	Columbus, OH, US	Collaborator	
Oh Young	Bang		MD	Samsung Medical Center, Departments of Neu	Seoul, South Korea	Site PI	
Jong-Won	Chung		MD, PhD	Samsung Medical Center, Departments of Neu	Seoul, South Korea	Collaborator	
Pyoung	Jeon		MD, PhD	Samsung Medical Center, Departments of Neu	Seoul, South Korea	Collaborator	
Keon Ha	Kim		MD, PhD	Samsung Medical Center, Departments of Neu	Seoul, South Korea	Collaborator	
Yun Jeong	Lim		RN	Samsung Medical Center, Departments of Neu	Seoul, South Korea	Collaborator	
Ji	Hoe Heo		MD	Yonsei University College of Medicine	Seoul, South Korea	Site PI	
Hyo Suk	Nam		MD, PhD	Yonsei University College of Medicine	Seoul, South Korea	Collaborator	
Young Dae	Kim		MD, PhD	Yonsei University College of Medicine	Seoul, South Korea	Collaborator	
HyungJong	Park		MD	Yonsei University College of Medicine	Seoul, South Korea	Collaborator	
In Gun	Hwang		MD	Yonsei University College of Medicine	Seoul, South Korea	Collaborator	
Wooseok	Ha		MD	Yonsei University College of Medicine	Seoul, South Korea	Collaborator	
Jin Kyo	Choi		MD	Yonsei University College of Medicine	Seoul, South Korea	Collaborator	
Kyungsun	Jeong		RN	Yonsei University College of Medicine	Seoul, South Korea	Collaborator	
Joung-Ho	Rha		MD	Inha University Hospital Neurology	Incheon, South Korea	Site PI	
Hee-Kwon	Park		MD	Inha University Hospital Neurology	Incheon, South Korea	Collaborator	
Cindy W.	Yoon		MD	Inha University Hospital Neurology	Incheon, South Korea	Collaborator	
Bo-Kyung	Kim		RN	Inha University Hospital Neurology	Incheon, South Korea	Collaborator	
Sung-Il	Sohn		MD	Dongsan Hospital, Keimyung University School	Daegu, South Korea	Site PI	
Chang-Hyun	Kim		MD	Dongsan Hospital, Keimyung University School	Daegu, South Korea	Site PI	
Joonsang	Yoo		MD	Dongsan Hospital, Keimyung University School	Daegu, South Korea	Collaborator	
Jeong-Ho	Hong		MD, PhD	Dongsan Hospital, Keimyung University School	Daegu, South Korea	Collaborator	
Hyungjong	Park		MD	Dongsan Hospital, Keimyung University School	Daegu, South Korea	Collaborator	
Sohyeon	Kim		MD	Dongsan Hospital, Keimyung University School	Daegu, South Korea	Collaborator	
Moonkyung	Choi		MD	Dongsan Hospital, Keimyung University School	Daegu, South Korea	Collaborator	
Hyoeun	Bae		MD	Dongsan Hospital, Keimyung University School	Daegu, South Korea	Collaborator	
Jun Seok	Lee		MD	Dongsan Hospital, Keimyung University School	Daegu, South Korea	Collaborator	

*Indicates required information. Only first name, last name, and suffix will appear in PubMed.

*First Name and Middle Initial(s)	*Last Name	*Suffix (eg, Jr, III)	Academic Degrees	Institution	Location (city, state/province, country)	Role or Contribution, eg, chair, principal investigator	Group (if more than 1 Group listed in the byline) and/or Subgroup (eg, Steering Committee)
Jae-Joon	Lee		MD	Dongsan Hospital, Keimyung University Schoo	Daegu, South Korea	Collaborator	
Go-Eun	Jun		BA (CRA)	Dongsan Hospital, Keimyung University Schoo	Daegu, South Korea	Collaborator	
Sujeong	Jeon		BA (CRA)	Dongsan Hospital, Keimyung University Schoo	Daegu, South Korea	Collaborator	
Bruce C. V.	Campbell		PhD	The Royal Melbourne Hospital, University of M	Parkville, Victoria, Austra	Site PI	
Peter J.	Mitchell		Mmed	The Royal Melbourne Hospital, University of M	Parkville, Victoria, Austra	Collaborator	
Nawaf	Yassi		PhD	The Royal Melbourne Hospital, University of M	Parkville, Victoria, Austra	Collaborator	
Stephen M.	Davis		MD	The Royal Melbourne Hospital, University of M	Parkville, Victoria, Austra	Collaborator	
Geoffrey A.	Donnan		MD	The Royal Melbourne Hospital, University of M	Parkville, Victoria, Austra	Collaborator	
Mark W.	Parsons		PhD	The Royal Melbourne Hospital, University of M	Parkville, Victoria, Austra	Collaborator	
Bernard	Yan		DMedSc	The Royal Melbourne Hospital, University of M	Parkville, Victoria, Austra	Collaborator	
Richard J.	Dowling		MBBS	The Royal Melbourne Hospital, University of M	Parkville, Victoria, Austra	Collaborator	
Steven J.	Bush		MBBS	The Royal Melbourne Hospital, University of M	Parkville, Victoria, Austra	Collaborator	
Teddy Y.	Wu		PhD	The Royal Melbourne Hospital, University of M	Parkville, Victoria, Austra	Collaborator	
Darshan G.	Shan		MBBS	The Royal Melbourne Hospital, University of M	Parkville, Victoria, Austra	Collaborator	
Henry	Zhao		MBBS	The Royal Melbourne Hospital, University of M	Parkville, Victoria, Austra	Collaborator	
Patrick	Salvaris		MBBS	The Royal Melbourne Hospital, University of M	Parkville, Victoria, Austra	Collaborator	
Fana	Alemseged		MD	The Royal Melbourne Hospital, University of M	Parkville, Victoria, Austra	Collaborator	
Felix C.	Ng		MBBS	The Royal Melbourne Hospital, University of M	Parkville, Victoria, Austra	Collaborator	
Cameron	Williams		MBBS	The Royal Melbourne Hospital, University of M	Parkville, Victoria, Austra	Collaborator	
Anna	Balabanski		MBBS	The Royal Melbourne Hospital, University of M	Parkville, Victoria, Austra	Collaborator	
Angela	dos Santos		MBBS	The Royal Melbourne Hospital, University of M	Parkville, Victoria, Austra	Collaborator	
Jo-Lyn	Ng		MBBS	The Royal Melbourne Hospital, University of M	Parkville, Victoria, Austra	Collaborator	
Amy	McDonald		BN	The Royal Melbourne Hospital, University of M	Parkville, Victoria, Austra	Collaborator	
David	Jackson		BN	The Royal Melbourne Hospital, University of M	Parkville, Victoria, Austra	Collaborator	
Jessica	Tsoleridis		BN	The Royal Melbourne Hospital, University of M	Parkville, Victoria, Austra	Collaborator	
Lauren	Pesavento		BN	The Royal Melbourne Hospital, University of M	Parkville, Victoria, Austra	Collaborator	
Timothy John	Kleinig		PhD	Royal Adelaide Hospital	Port Road, Adelaide Sout	Site PI	
Roy	Drew		B Nurs	Royal Adelaide Hospital	Port Road, Adelaide Sout	Collaborator	
Jennifer	Cranefield		M Nurs	Royal Adelaide Hospital	Port Road, Adelaide Sout	Collaborator	
Rebecca	Scroop		MBBS	Royal Adelaide Hospital	Port Road, Adelaide Sout	Collaborator	
Lavenia	Cagi		MBBS	Royal Adelaide Hospital	Port Road, Adelaide Sout	Collaborator	
Jackson	Harvey		MBBS	Royal Adelaide Hospital	Port Road, Adelaide Sout	Collaborator	
Michael J.	Waters			Royal Adelaide Hospital	Port Road, Adelaide Sout	Collaborator	

*Indicates required information. Only first name, last name, and suffix will appear in PubMed.

***First Name and Middle Initial(s)**	***Last Name**	***Suffix (eg, Jr, III)**	Academic Degrees	Institution	Location (city, state/province, country)	Role or Contribution, eg, chair, principal investigator	Group (if more than 1 Group listed in the byline) and/or Subgroup (eg, Steering Committee)
John	Thornton		MD	Beaumont Hospital	Dublin, Ireland	Site PI	
Robert P.	Brennan		MD RCSI	Beaumont Hospital	Dublin, Ireland	Collaborator	
Alan	O'Hare		MD	Beaumont Hospital	Dublin, Ireland	Collaborator	
Sarah	Power		PhD	Beaumont Hospital	Dublin, Ireland	Collaborator	
David JP	Williams		PhD RCSI	Beaumont Hospital	Dublin, Ireland	Collaborator	
Karl	Boyle		MD	Beaumont Hospital	Dublin, Ireland	Collaborator	
Barry J.	Moynihan		MD RCSI	Beaumont Hospital	Dublin, Ireland	Collaborator	
Ann-Marie	Liddy		MRCPI, PhD	Beaumont Hospital	Dublin, Ireland	Collaborator	
Margaret	Large		Bsc, RCSI	Beaumont Hospital	Dublin, Ireland	Collaborator	
Ailbhe	Cullen		RGN, RSCN, MSc RCSI	Beaumont Hospital	Dublin, Ireland	Collaborator	
Roisin	Walsh		RGN, Hdip, Pgrad, MSc RCSI	Beaumont Hospital	Dublin, Ireland	Collaborator	
Emma	Martin		Bsc, Pgdip	Beaumont Hospital	Dublin, Ireland	Collaborator	
Julie	Lynch		RGN, MSc	Beaumont Hospital	Dublin, Ireland	Collaborator	
Sinéad	McElroy			Beaumont Hospital	Dublin, Ireland	Collaborator	
Seán	Murphy		MD	Mater Misericordiae University Hospital	Dublin, Ireland	Site PI	
Sarah A.	Coveney		MRCPI	Mater Misericordiae University Hospital	Dublin, Ireland	Collaborator	
Caroline	Deegan			Mater Misericordiae University Hospital	Dublin, Ireland	Collaborator	
Gillian	Horgan		BSc	Mater Misericordiae University Hospital	Dublin, Ireland	Collaborator	
Peter	Kelly		MD	Mater Misericordiae University Hospital	Dublin, Ireland	Collaborator	
Aoife	Laffan		MD	Mater Misericordiae University Hospital	Dublin, Ireland	Collaborator	
Yudy	Llamas-Osorio		MD	Mater Misericordiae University Hospital	Dublin, Ireland	Collaborator	
Catherine T.	Lynch		BSN	Mater Misericordiae University Hospital	Dublin, Ireland	Collaborator	
Peter J.	Mac Mahon		FFR(RCSI)	Mater Misericordiae University Hospital	Dublin, Ireland	Collaborator	
Michael	Marnane		MB PhD	Mater Misericordiae University Hospital	Dublin, Ireland	Collaborator	
John J.	McCabe		MRCPI	Mater Misericordiae University Hospital	Dublin, Ireland	Collaborator	
Paul A.	Burns		MB	Royal Victoria Hospital	Belfast, Northern Ireland	Site PI	
Suzanne	Tauro			Royal Victoria Hospital	Belfast, Northern Ireland	Collaborator	
Sarah	Cuddy			Royal Victoria Hospital	Belfast, Northern Ireland	Collaborator	

Supplemental Online Content: Nonauthor Collaborators

*Indicates required information. Only first name, last name, and suffix will appear in PubMed.

***First Name and Middle Initial(s)**	***Last Name**	***Suffix (eg, Jr, III)**	Academic Degrees	Institution	Location (city, state/province, country)	Role or Contribution, eg, chair, principal investigator	Group (if more than 1 Group listed in the byline) and/or Subgroup (eg, Steering Committee)
Ian	Rennie			Royal Victoria Hospital	Belfast, Northern Ireland	Collaborator	
Graham	Smyth			Royal Victoria Hospital	Belfast, Northern Ireland	Collaborator	
Peter	Flynn			Royal Victoria Hospital	Belfast, Northern Ireland	Collaborator	
Ivan	Wiggam			Royal Victoria Hospital	Belfast, Northern Ireland	Collaborator	
Enda	Kerr			Royal Victoria Hospital	Belfast, Northern Ireland	Collaborator	
Patricia	Gordon			Royal Victoria Hospital	Belfast, Northern Ireland	Collaborator	
Patricia	Fearon			Royal Victoria Hospital	Belfast, Northern Ireland	Collaborator	
Geralt	Roberts			Royal Victoria Hospital	Belfast, Northern Ireland	Collaborator	
Cathy	Patterson			Royal Victoria Hospital	Belfast, Northern Ireland	Collaborator	
Karen	Adams			Royal Victoria Hospital	Belfast, Northern Ireland	Collaborator	
Brian	Wells			Royal Victoria Hospital	Belfast, Northern Ireland	Collaborator	
Margaret	McFarland			Royal Victoria Hospital	Belfast, Northern Ireland	Collaborator	
Tiago	Moreira		MD, PhD	Department of Clinical Neuroscience, Karolins	Stockholm, Sweden	Collaborator	
Christina	Sjöstrand		MD, PhD	Department of Clinical Neuroscience, Karolins	Stockholm, Sweden	Collaborator	
Michael	Mazya		MD, PhD	Department of Clinical Neuroscience, Karolins	Stockholm, Sweden	Collaborator	
Håkan	Almqvist		MD	Department of Clinical Neuroscience, Karolins	Stockholm, Sweden	Collaborator	
Nils	Wahlgren		MD, PhD	Department of Clinical Neuroscience, Karolins	Stockholm, Sweden	Collaborator	
Anna	Steinberg		MD, PhD	Department of Clinical Neuroscience, Karolins	Stockholm, Sweden	Collaborator	
Charith	Cooray		MD, PhD	Department of Clinical Neuroscience, Karolins	Stockholm, Sweden	Collaborator	
Einar	Eriksson		MD, PhD	Department of Clinical Neuroscience, Karolins	Stockholm, Sweden	Collaborator	
Magnus	Thorén		MD, PhD	Department of Clinical Neuroscience, Karolins	Stockholm, Sweden	Collaborator	
Boris	Keselman		MD, PhD	Department of Clinical Neuroscience, Karolins	Stockholm, Sweden	Collaborator	
Niaz	Ahmed		MD, PhD	Department of Clinical Neuroscience, Karolins	Stockholm, Sweden	Collaborator	

Supplemental Online Content: Nonauthor Collaborators

*Indicates required information. Only first name, last name, and suffix will appear in PubMed.

*First Name and Middle Initial(s)	*Last Name	*Suffix (eg, Jr, III)	Academic Degrees	Institution	Location (city, state/province, country)	Role or Contribution, eg, chair, principal investigator	Group (if more than 1 Group listed in the byline) and/or Subgroup (eg, Steering Committee)
Åke	Holmberg			Department of Clinical Neuroscience, Karolins	Stockholm, Sweden	Collaborator	
Maria C.	Axelsson			Department of Clinical Neuroscience, Karolins	Stockholm, Sweden	Collaborator	
Annika	Berglund		PhD	Department of Clinical Neuroscience, Karolins	Stockholm, Sweden	Collaborator	
Amir	Golsari		MD	University Medical Center Hamburg-Eppendor	Hamburg, Germany	Collaborator	
Julia	Hoppe		MD	University Medical Center Hamburg-Eppendor	Hamburg, Germany	Collaborator	
Milani	Deb-Chatterji		MD	University Medical Center Hamburg-Eppendor	Hamburg, Germany	Collaborator	
Bastian	Cheng		MD	University Medical Center Hamburg-Eppendor	Hamburg, Germany	Collaborator	
Ewgenia	Barow		MD	University Medical Center Hamburg-Eppendor	Hamburg, Germany	Collaborator	
Eckhard	Schlemm		MD	University Medical Center Hamburg-Eppendor	Hamburg, Germany	Collaborator	
Iris	Lettow		MD	University Medical Center Hamburg-Eppendor	Hamburg, Germany	Collaborator	
Märit	Jensen		MD	University Medical Center Hamburg-Eppendor	Hamburg, Germany	Collaborator	
Dagmar	Otto			University Medical Center Hamburg-Eppendor	Hamburg, Germany	Collaborator	
Kirsten	Jaramillo			University Medical Center Hamburg-Eppendor	Hamburg, Germany	Collaborator	
Hannes	Appelbohm			University Medical Center Hamburg-Eppendor	Hamburg, Germany	Collaborator	
Jens	Fiehler		MD	University Medical Center Hamburg-Eppendor	Hamburg, Germany	Collaborator	
Maxim	Bester		MD	University Medical Center Hamburg-Eppendor	Hamburg, Germany	Collaborator	
Michael	Schönfeld		MD	University Medical Center Hamburg-Eppendor	Hamburg, Germany	Collaborator	
Caspar	Brekenfeld		MD	University Medical Center Hamburg-Eppendor	Hamburg, Germany	Collaborator	
Brigette	Holst		MD	University Medical Center Hamburg-Eppendor	Hamburg, Germany	Collaborator	
Ginette	Wortmann			University Medical Center Hamburg-Eppendor	Hamburg, Germany	Collaborator	
Friederike	Koch			University Medical Center Hamburg-Eppendor	Hamburg, Germany	Collaborator	
Johannes C.	Gerber		MD	University Hospital Carl Gustav Carus at the Te	Dresden, Germany	Collaborator	
Andrij	Abramyuk		MD	University Hospital Carl Gustav Carus at the Te	Dresden, Germany	Collaborator	
Daniel	Kaiser		MD	University Hospital Carl Gustav Carus at the Te	Dresden, Germany	Collaborator	
Simon	Winzer		MD	University Hospital Carl Gustav Carus at the Te	Dresden, Germany	Collaborator	
Alexandra	Prakapenia		MD	University Hospital Carl Gustav Carus at the Te	Dresden, Germany	Collaborator	
Lars-Peder	Pallesen		MD	University Hospital Carl Gustav Carus at the Te	Dresden, Germany	Collaborator	
Timo	Siepmann		MD	University Hospital Carl Gustav Carus at the Te	Dresden, Germany	Collaborator	
Kristian	Barlinn		MD	University Hospital Carl Gustav Carus at the Te	Dresden, Germany	Collaborator	
Kathrin	Haase		RN	University Hospital Carl Gustav Carus at the Te	Dresden, Germany	Collaborator	
Angela	Sauer		RN	University Hospital Carl Gustav Carus at the Te	Dresden, Germany	Collaborator	
Marios-Nikos	Psychogios		MD	University Medical Center Göttingen	Göttingen, Germany	Site PI	
Jan	Liman		MD	University Medical Center Göttingen	Göttingen, Germany	Collaborator	

*Indicates required information. Only first name, last name, and suffix will appear in PubMed.

*First Name and Middle Initial(s)	*Last Name	*Suffix (eg, Jr, III)	Academic Degrees	Institution	Location (city, state/province, country)	Role or Contribution, eg, chair, principal investigator	Group (if more than 1 Group listed in the byline) and/or Subgroup (eg, Steering Committee)
Alex	Brehm		MD	University Medical Center Göttingen	Göttingen, Germany	Collaborator	
Volker	Maus		MD	University Medical Center Göttingen	Göttingen, Germany	Collaborator	
Nicole	Hollstein			University Medical Center Göttingen	Göttingen, Germany	Collaborator	
Annika	Reinke			University Medical Center Göttingen	Göttingen, Germany	Collaborator	
Gustav	Neitz			University Medical Center Göttingen	Göttingen, Germany	Collaborator	
Marlena	Schnieder		MD	University Medical Center Göttingen	Göttingen, Germany	Collaborator	
Margret	Schwarz			University Medical Center Göttingen	Göttingen, Germany	Collaborator	
Allam	Ibrahim		MD	University Medical Center Göttingen	Göttingen, Germany	Collaborator	
Julia	Müller			University Medical Center Göttingen	Göttingen, Germany	Collaborator	
Stefanie	Meister			University Medical Center Göttingen	Göttingen, Germany	Collaborator	
Berit	Bäumle			University Medical Center Göttingen	Göttingen, Germany	Collaborator	
René	Chapot		MD	Alfred Krupp Krankenhaus Hospital, Departme	Essen, Germany	Site PI	
Sara	Pilgram-Pastor		MD	Alfred Krupp Krankenhaus Hospital, Departme	Essen, Germany	Collaborator	
Marta	Wallocha		MD	Alfred Krupp Krankenhaus Hospital, Departme	Essen, Germany	Collaborator	
Michael	Stauder		MD	Alfred Krupp Krankenhaus Hospital, Departme	Essen, Germany	Collaborator	
Ekin	Celik		MD	Alfred Krupp Krankenhaus Hospital, Departme	Essen, Germany	Collaborator	
Paul	Stracke		MD	Alfred Krupp Krankenhaus Hospital, Departme	Essen, Germany	Collaborator	
Nicole	Müller			Alfred Krupp Krankenhaus Hospital, Departme	Essen, Germany	Collaborator	
Ralph	Weber		MD	Alfred Krupp Krankenhaus Hospital, Departme	Essen, Germany	Collaborator	
R.	Veltkamp		MD	Alfred Krupp Krankenhaus Hospital, Departme	Essen, Germany	Collaborator	
Simon	Nagel		MD	University Hospital Heidelberg	Heidelberg, Germany	Site PI	
Peter Arthur	Ringleb		MD	University Hospital Heidelberg	Heidelberg, Germany	Collaborator	
Miriam	Heyse		MD	University Hospital Heidelberg	Heidelberg, Germany	Collaborator	
Hemasse	Amiri		MD	University Hospital Heidelberg	Heidelberg, Germany	Collaborator	
Sibu	Mundiyanapurath		MD	University Hospital Heidelberg	Heidelberg, Germany	Collaborator	
Min	Chen		MD	University Hospital Heidelberg	Heidelberg, Germany	Collaborator	
Elisabeth	Beyrle			University Hospital Heidelberg	Heidelberg, Germany	Collaborator	
Perdita	Beck			University Hospital Heidelberg	Heidelberg, Germany	Collaborator	
Markus Alfred	Möhlenbruch		MD	University Hospital Heidelberg	Heidelberg, Germany	Collaborator	
