# Supplementary material for: Mediation of Age and Thrombectomy Outcome by Neuroimaging Markers of Frailty in Patients With Stroke
Source: JAMA Netw Open. 2024 Jan 2;7(1):e2349628. doi: 10.1001/jamanetworkopen.2023.49628 (PMC10762575; doi:10.1001/jamanetworkopen.2023.49628)
Supplement: Supplement 3. — Data Sharing Statement [file jamanetwopen-e2349628-s003.pdf]

Data Sharing Statement

Benali. Mediation of Age and Thrombectomy Outcome by Neuroimaging Markers of Frailty in Patients With Stroke. *JAMA Netw Open*. Published December 28, 2023.
doi:10.1001/jamanetworkopen.2023.49628

Data
Data available: No

Additional Information
Explanation for why data not available: The ESCAPE-NA1 data are not currently publicly available for distribution, but a future public data set may be made available. Researchers interested in the data are asked to contact the corresponding author with a proposal.
